# Rescuing Botany: using citizen-science and mobile apps in the classroom and beyond

**DOI:** 10.1038/s44185-023-00011-9

**Published:** 2023-03-01

**Authors:** Sergio Chozas, Alice Nunes, Helena C. Serrano, Fernando Ascensão, Susana Tapia, Cristina Máguas, Cristina Branquinho

**Affiliations:** 1 Centre for Ecology, Evolution and Environmental Changes (cE3c) & Global Change and Sustainability Institute (CHANGE), Faculdade de Ciências da Universidade de Lisboa, Lisbon, Portugal; 2 Sociedade Portuguesa de Botânica (SPBotânica), Lisbon, Portugal

**Keywords:** Ecology, Plant sciences

## Abstract

Biodiversity is declining due to the impact of human activities. However, public awareness of the biodiversity crisis is low, particularly for plants, creating a barrier to engage with conservation programs. In this perspective, we show how citizen science and mobile apps can be used as educational tools to raise awareness about plant biodiversity among students and the general public. We examine the outcomes of three Bachelor of Science activities as well as two informal education initiatives. We discuss the potential of these approaches as educational and outreach tools. Our results show that citizen science and mobile apps are excellent tools for engaging society in biodiversity conservation and environmental issues.

## Introduction

Global biodiversity has been dramatically declining over the last decades^1–4^. The current biodiversity crisis is primarily driven by human-induced factors, the most serious of which are land-use change, habitat fragmentation, and climate change^5^. While global public awareness of climate change matters is high^6,7^, public recognition of biodiversity loss has, historically, been low^8^. The understanding of biodiversity concepts highly varies among countries and social groups^9–11^: in Nigeria, the biodiversity concept was known of 20.5% of non-professional Nigerians (with basic education or no formal training) while among 88.8% of professionals with tertiary education, it reached 88.8%; 60% of participants in a study in Switzerland had never heard the term biodiversity and Chinese farmers in another pilot study have never heard about biodiversity. In the European Union, the global leader of the environmental movement on both the political and discursive levels^12,13^, in 2018, 71% of EU citizens had heard of biodiversity, but only around 41% of these knew what biodiversity meant^14^. This illiteracy is a significant constraint for conservation strategies because the development and success of actions to halt and reverse biodiversity loss strongly rely on public support^15^.

If general awareness of biodiversity loss is low, knowledge about plant diversity is even lower^16^. Plants have traditionally been overlooked, and expressions such as "plant blindness", defined as a human tendency to ignore plant species^17^, perfectly illustrate the situation in terms of plant conservation. And yet, current estimates suggest that two out of five plant species are threatened with extinction^18^. Moreover, plants play a crucial role in the world ecosystems by providing habitat, shelter, oxygen, and food, including for humans^19^. Local community support boosts the effectiveness of biodiversity conservation actions^20–22^. However, how biodiversity is perceived and the benefits it provides to local populations have a significant influence on this support^23^. Therefore, stopping the loss of plant biodiversity and the impact it has on ecosystem health and human well-being must also strive to raise public awareness on the importance of plant conservation^24^.

A big challenge, however, is to engage people with conservation. Nowadays, in a world where a large part of the human population lives in urban areas, the contact of people with nature is declining. This is a trend that will be even more accentuated in the future^25^. Perhaps society’s interest in plants is decreasing because of limited exposure to plants in daily lives, schools, and work. However, by critically examining our roles as plant scientists and educators, we realize that there are probably things we could, and should, do differently. New strategies to connect people to nature are required to spark people’s interest in and knowledge of plants. Citizen science programs and mobile applications (apps) are noteworthy initiatives that are helping to achieve this goal.

Citizen science is defined as the general public involvement in scientific research activities and currently is a mainstream approach to collect information and data on a wide range of scientific subjects^26,27^. The development of mobile technologies and the widespread use of smartphones have boosted citizen science and enabled the development of mobile apps, which are digital tools that integrate, in real-time, data from multiple sources^28^.

The goal of this article is to show how citizen science and mobile apps can be used as educational tools to raise awareness about plant biodiversity and conservation among the general public. We focused on formal education activities, at the Bachelor of Science (BSc) level, that were designed to collect data on various aspects of plant community and functional ecology. We also present the outcomes of two informal education initiatives that used citizen science to gather data on the distribution of plant diversity. We discuss these activities and results in light of their potential to engage the public into biodiversity conservation, and as educational and outreach tools.

### Formal education: University

During the COVID-19 pandemic (2021), Ecology practical classes of the Bologna Bachelor Degree in Biology (Faculty of Sciences of the University of Lisbon) had to be adapted to remote learning. Fortunately, during the States of Emergency imposed by the Portuguese Government, citizens were allowed to take brief walks. Taking advantage of citizen’s ability to briefly travel outdoors, we created three activities for students, as alternatives to those typically carried out in the classroom/campus, which we describe below.

### Activity 1—Analysis of the impact of disturbance on plant diversity in grasslands

The objective of this activity was for students to explore the impact of disturbance and site attributes (such as soil type) on the diversity of the herbaceous plant community and its associated pollinators. This was undertaken in grasslands located near their homes, within walking distance (due to COVID lockdown movement restrictions). To achieve this goal, we developed a comprehensive sampling protocol that included methods for (i) selecting and characterizing sampling sites based on the level of human perturbation, (ii) soil characterization, (iii) sampling, identifying, and registering plants using the iNaturalist/Biodiversity4All platform and Flora-on web (Box 1), and (iv) pollinator sampling (Supplementary Data 1). To ensure accurate plant and pollinators identification, all observations were verified by professors responsible for each topic.

First, each student chose one sampling site and teachers, using photographs, classified all sites regarding their perturbation level (low, medium, and high). Then, using the sampling protocol, students were invited to study different aspects of their sampling site, *in loco* or at their homes. Soil samples were analysed using simple methods and available household instruments (such as plastic cups, kitchen scale, and oven). Students were introduced to soil biodiversity as well as soil parameters (humidity, texture, structure, infiltration and draining) during the remote classes. Plants were sampled using a home-made 1 m^2^ quadrat. All species within were counted and identified to the lowest taxonomic level possible, using the mentioned apps and website. Before plant sampling, students were also asked to count and identify pollinators within their quadrats (broad taxonomic groups, bees, butterflies, flies, beetles) for 5 min, again using the apps to aid identification.

Following field sampling, students were asked to calculate two taxonomic indices of plant communities. These included species richness, which measures the number of different species that occur in a sample, and the Simpson Diversity Index, which evaluates the probability that two individuals randomly selected from a sample will belong to the same species. Students also calculated functional diversity indices such as Functional Richness and Functional Dissimilarity, since functional diversity explores functional differences between species and how these differences reflect and affect the interactions with the environment and with other species^29^. Then, students assessed the relation between these indices and perturbation level. They analysed several functional traits of plants that are likely to respond to local perturbation (e.g., height, leaf size). Finally, they attempted to relate plant indices with the occurrence of pollinators.

**Fig. 1:**
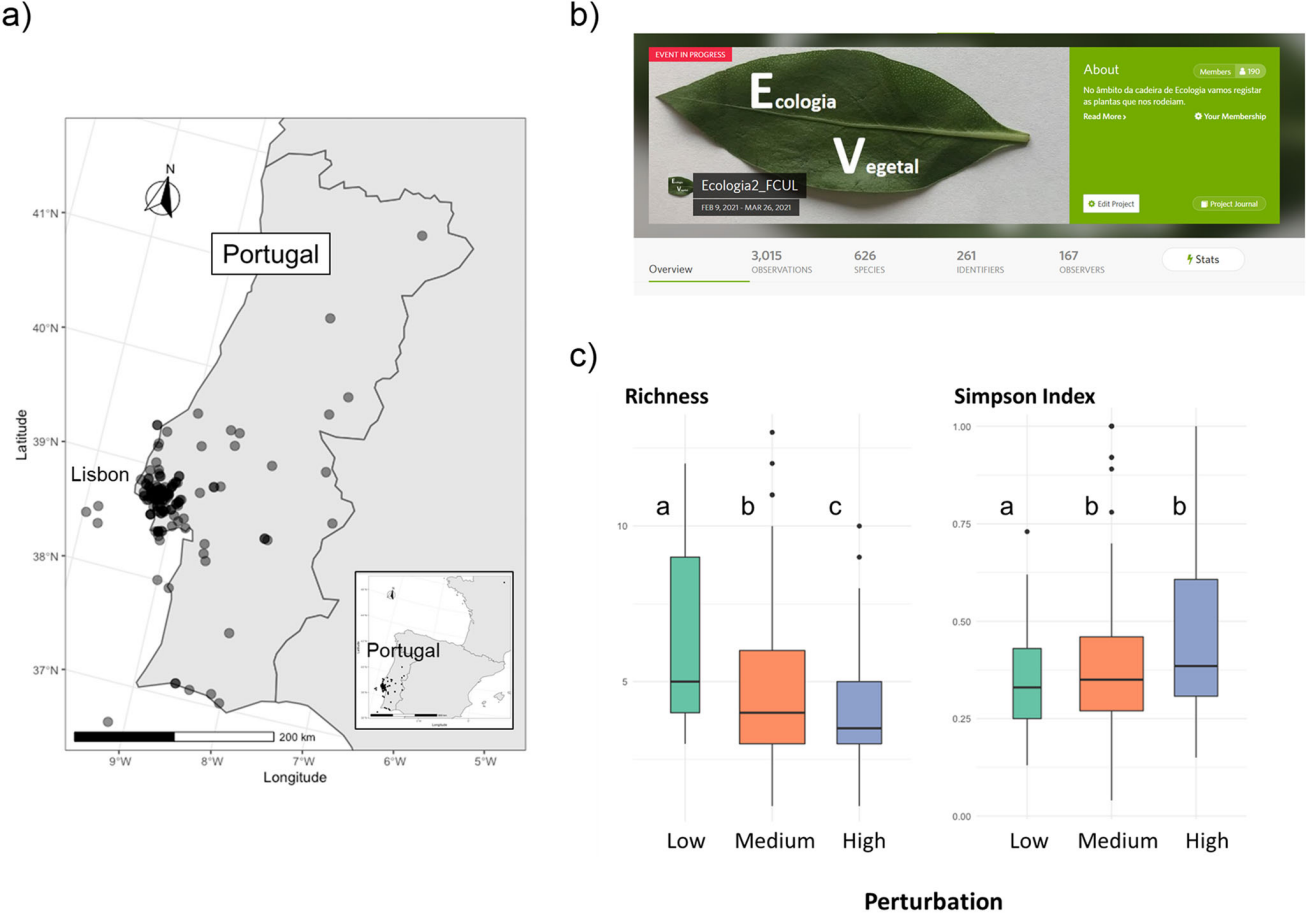
Analysis of the impact of disturbance on plant diversity in grasslands. **a** Location of grasslands sampled; **b** Banner and overview of main results of the project created in the platform iNaturalist/Biodiversity4All to register the sampled species; **c** Boxplots include data of the taxonomic diversity indices (plant species richness and Simpson Diversity Index) of sampled grasslands at three different perturbation levels: low, medium and high. Central lines represent median values, box limits indicate the upper and lower quartiles, whiskers correspond to 1.5 × the interquartile range above and below the upper and lower quartiles and points are the outliers. Boxplots with different letters indicate statistically significant differences among perturbation levels based on multiple pairwise comparisons.

Overall, students sampled 147 grasslands that were affected by low (*n* = 17); medium (*n* = 86) and high (*n* = 40) levels of perturbation, scattered across mainland Portugal (Fig. 1a). In total, 3015 observations corresponding to 543 species of plant and 88 of insects (Fig. 1b) were registered in the iNaturalist/Biodiversity4All project Ecologia2_FCUL, created specifically to record all of the diversity data associated with this activity. Other registered taxa included six species of molluscs and 13 of arachnids, and other occasional soil macrofauna.

The results showed that the number of species (richness) decreased consistently with the level of perturbation. Simpson Diversity Index values increased, indicating low diversity values in highly perturbed herbaceous plant communities (Fig. 1c). Results revealed a trend towards an increase in the proportion of species with lower stature as perturbation increased. However, with no clear relationship with either biodiversity or perturbation. Finally, results indicated no clear relation of pollinator abundance or richness with plant richness and diversity, although field records relate a lower number of pollinators as wind intensity increased. In fact, pollinator sampling is extremely weather sensitive, which may have contributed to the lack of consistent relationships between pollinator diversity and perturbation.

Box 1 Citizen science platforms and apps used for formal and informal educational activities iNaturalist (https://www.inaturalist.org/home): is a social network of naturalists, citizen scientists, and biologists that is based on mapping and sharing biodiversity observations. They describe themselves as “an online social network of people sharing biodiversity information in order to help each other learn about nature”. iNaturalist may be accessed via website or mobile app. Records are validated by the iNaturalist community. Observations reached approximately 110 million as of July 2022. This app allows the development of both open-access and registration-restricted projects. BioDiversity4All (https://www.biodiversity4all.org/) is a Portuguese biodiversity citizen science platform created by the Biodiversity for All Association. This platform was founded in 2010 and is currently linked to the “iNaturalist” network^43^. All the projects presented in this article were developed on the Biodiversity4All platform.

Flora-on (https://flora-on.pt/): this portal contains occurrence data of vascular plants from the Portuguese flora collected by project collaborators (over 575,000 records as of July 2022). Flora-on was created by the Botanical Society of Portugal (SPBotânica), a Portuguese association devoted to the promotion and study of botany in Portugal. Botanists and naturalists provide most of the data, but occasional contributors are welcomed. Records are supervised by the portal editors, ensuring the dataset’s quality level. The portal includes stunning images of leaves, flowers, fruits, and other plant parts for 2299 of the 3300 taxa occurring in Portugal^44^. Additionally, the portal includes a powerful search engine that allows geographical, morphological, and taxonomical searches.

LeafBite (https://zoegp.science/leafbyte): is a free, open-source iPhone app that measures total leaf area as well as consumed leaf area when herbivory is present^45^.

Leaf-IT is a free and simple Android app created for scientific purposes. It was designed to measure leaf area under challenging field conditions. It has simple features for area calculation and data output, and can be used for ecological research and education^46^.

### Activity 2—Leaf trait assessment of shrub and tree species

Students were asked to assess three leaf traits Specific leaf area (SLA), Specific leaf mass (LMA), and Leaf Water Content (LWC) of two or three shrub or tree species. Each species should ideally fall into one of three functional groups known for their water adaptations, namely Hydrophytes, Mesophytes and Xerophytes. Students were challenged to choose charismatic Mediterranean species that grew nearby, such as *Olea europaea, Nerium oleander or Phillyrea angustifolia*. Alternatively, they could take the “*Quercus* challenge”, which involved ranking the Portuguese oak species based on their drought tolerance. A detailed protocol was developed to assist students for this purpose (Supplementary Data 2). In this protocol was demonstrated how to calculate the leaf area using the LeafBite and Leaf-IT apps (Box 1).

**Fig. 2:**
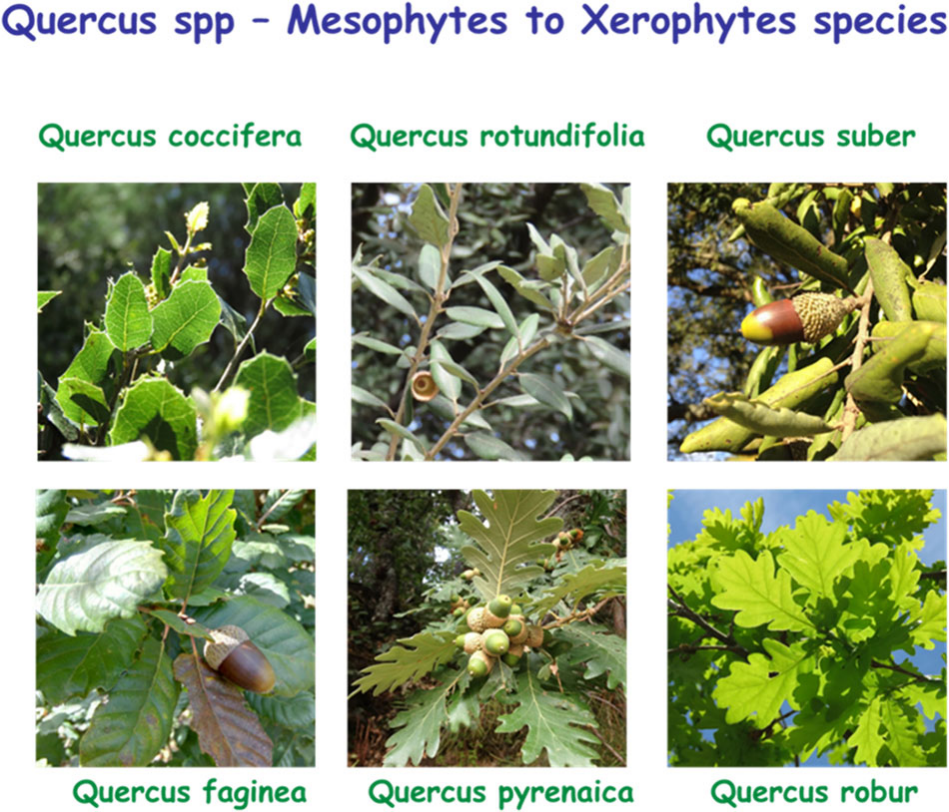
Leaf trait assessment of shrub and tree species: *Quercus* challenge. Classification of Portuguese oak species regarding their drought tolerance (higher tolerance, left-up, lower tolerance right-down).

The students calculated the SLA, LMA, and LWC of a total of 104 species (Supplementary Data 3) belonging to the main functional groups under study. Regarding the “*Quercus* challenge”, they were able to classify the six most representative oak species in Portugal and confirm the relationship among these indices and their tolerance to drought (Fig. 2).

One of the students, accomplished to present his own learning experience related to these activities at the XXIII Conference of the Environmental Research Network of Portuguese-speaking Nations - REALP, under the title “Plant Ecology during Confinement - A Digital Approach”.

### Activity 3—Evaluating the impact on the biodiversity of lawn management at the University of Lisbon campus

Although, after the lockdown, practical classes returned to the laboratories and the field in 2021/22, we continued to use the iNaturalist/Biodiversity4All platform and the Flora-on website for biodiversity registering and identification, because of the success of the activities, as evidenced by the positive comments we received from students.

The goal of this activity was to study the impact of lawn management on plant diversity and pollination on the University of Lisbon campus. To accomplish this, the students described the herbaceous communities and pollinators on four lawns (named C8, RL, RR, and TT) that had different management practices (mowing and irrigation). A comprehensive document with sampling guidelines was developed (Supplementary Data 4).

**Fig. 3:**
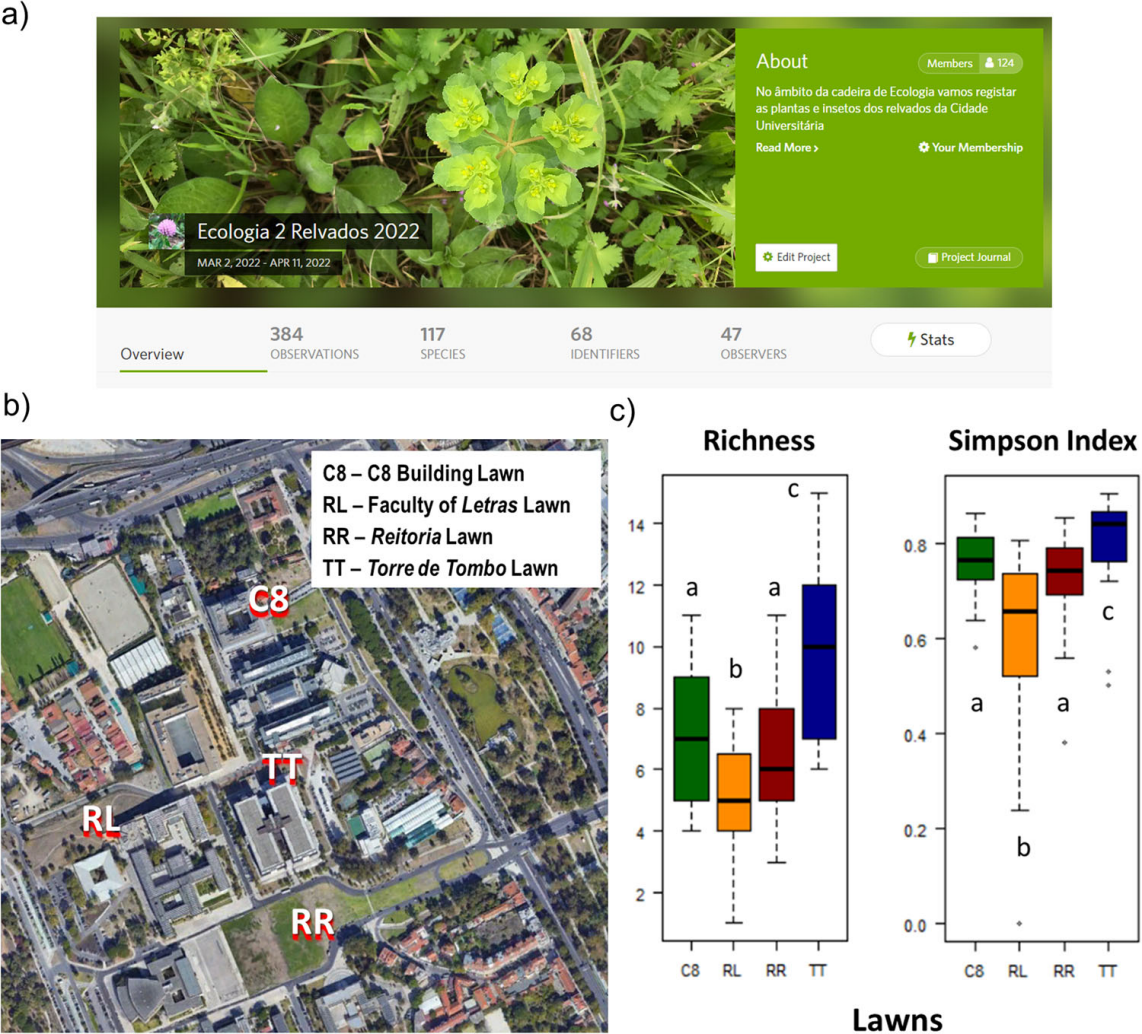
Evaluating the impact on the biodiversity of lawn management at the University of Lisbon campus. **a** Banner and overview of main results of the project Ecologia 2 Relvados created in the platform iNaturalist/BioDiversity4All to register the sampled species; **b** Location of the lawns sampled in the Campus of the University of Lisbon; **c** Boxplots include data of the taxonomic diversity indices (plant species richness and Simpson Diversity Index) of sampled grasslands. Central lines represent median values, box limits indicate the upper and lower quartiles, whiskers correspond to 1.5 × the interquartile range above and below the upper and lower quartiles and points are the outliers. Boxplots with different letters indicate statistically significant differences among lawns based on multiple pairwise comparisons.

The project Ecologia 2 Relvados 2022 registered 100 plant and 17 pollinator species (Fig. 3a). Given that the sampling took place during a cold and rainy week, which limited pollinator activity, the low number of pollinators registered was expectable (Lawson and Rands 2019). Following these analyses, the TT lawn (Fig. 3b), which had low levels of mowing and no watering, showed a significantly higher value of diversity, indicating it had the best management strategy for these systems (Fig. 3c), if the goal is to increase biodiversity.

### Informal education: BioBlitzes

**Fig. 4:**
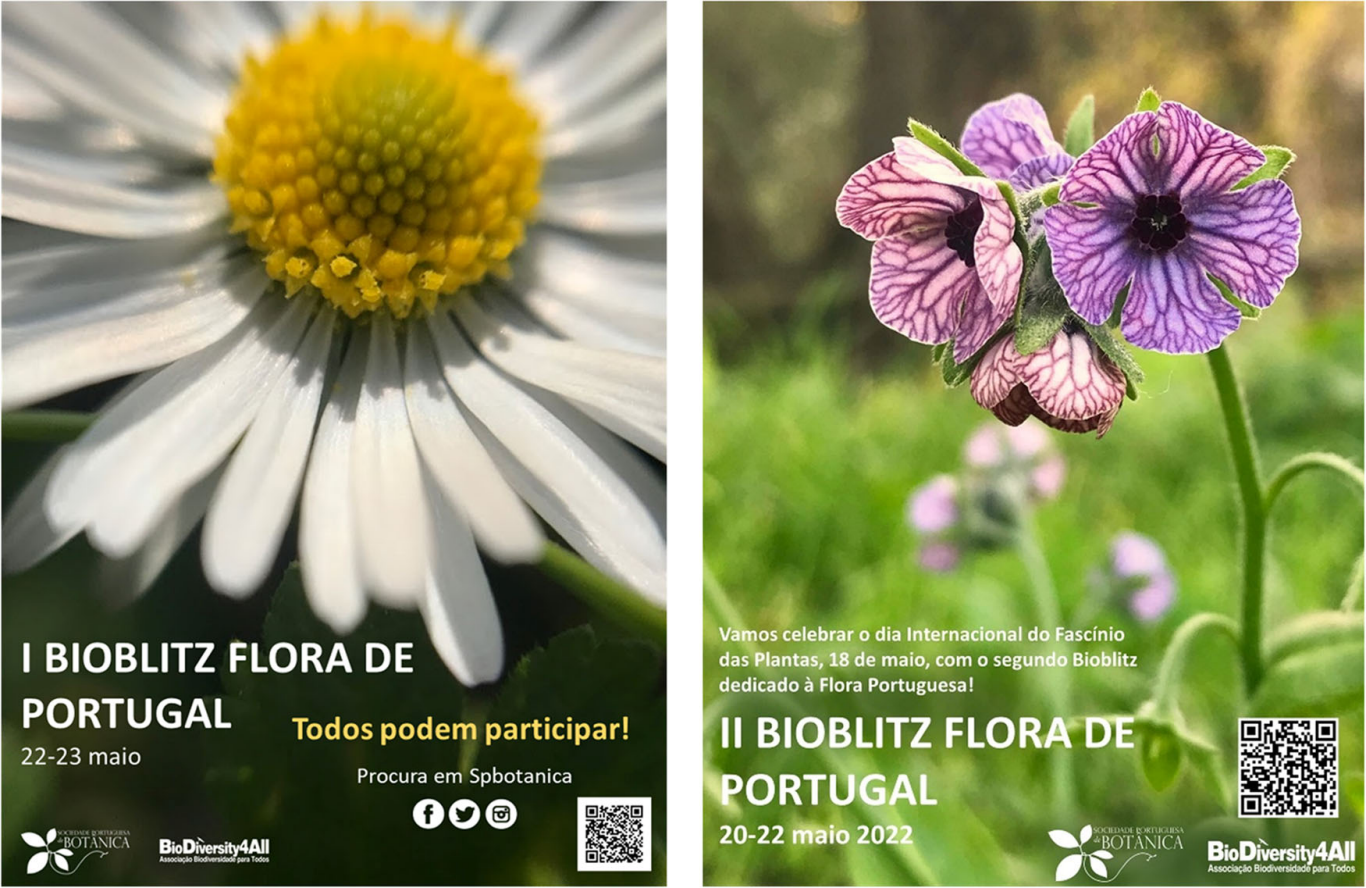
Bioblitz I & II – Flora of Portugal. Posters created for the promotion of the two Flora of Portugal Bioblitzes.

Intense biological surveys known as “BioBlitz” are carried out to record all organisms found in certain locations, such as cities, protected areas, or even entire countries. They are being used all over the world to collect and share georeferenced biodiversity data^30^. We developed two Plant Bioblitzes based on the BioDiversity4All/iNaturalist and Flora-on platforms. Social media, such as Facebook, Instagram, and Twitter, were used to promote these events and engage citizens (Fig. 4). The BioBlitzes were developed by SPBotânica in collaboration with BioDiversity4All.

### Bioblitz I & II - Flora of Portugal

The celebration of Fascination of Plants Day (18th of May) served as the backdrop for the organization of two-weekend Bioblitzes: Bioblitz Flora of Portugal I and Bioblitz Flora of Portugal II.

**Fig. 5:**
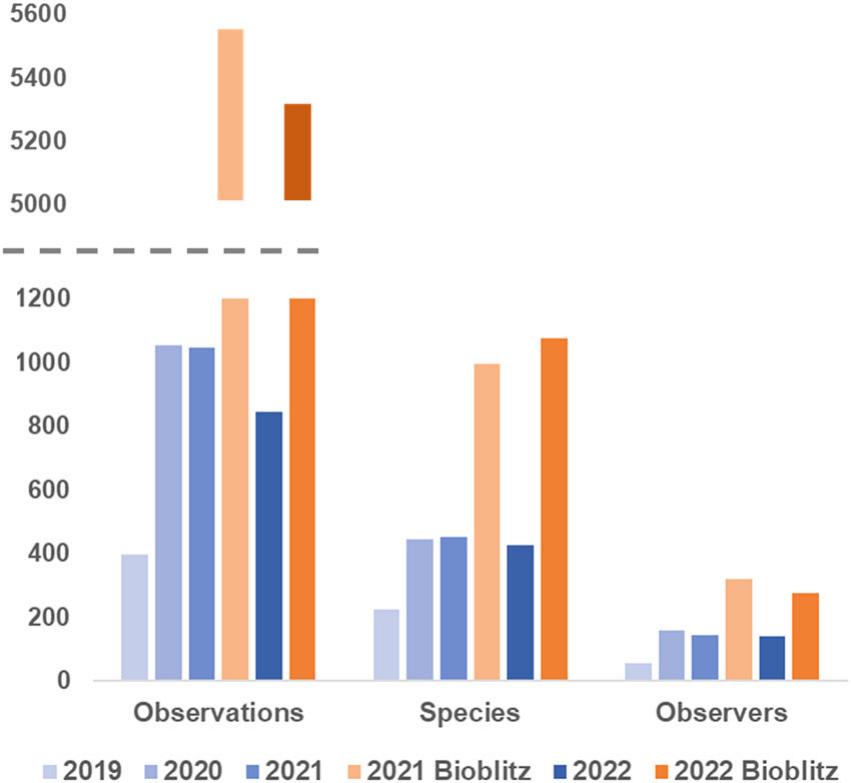
Number of observations, species and observers registered on the BioDiversity4All/iNaturalist platform over equivalent weekends in 2019, 2020, 2021, and 2022. Numbers for 2021 and 2022 correspond to the weekends in which Bioblitzes I & II - Flora of Portugal were conducted, as well as previous ones.

In 2021, the Bioblitz was solely focused on project members, which meant that only those who had voluntarily joined the initiative could participate. In total, the 119 project members registered 4234 observations of 890 plant species. In contrast, the 2022 Bioblitz was an open project (no registration required). In total, the 323 observers made 6547 records of 1198 species. To evaluate the impact of the Bioblitz events, we compared the data registered in BioDiverstiy4All during the weekends of both events (2021 and 2022) with (i) the data registered in the platform during the equivalent weekends of 2019 and 2020 and (ii) also during the weekends before both Bioblitzes. The number of species, observations, and observers increased significantly from 2019 to 2020, 2021, and 2022, but, when comparing values from 2020 with 2021 and 2022, this rise was only verified during the Bioblitz weekends, proving the importance of Bioblitzes in this increase (Fig. 5).

## Discussion

According to our experience, activities involving citizen science platforms and mobile apps have a high potential to be used as tools in formal education while also contributing to scientific research. This approach allowed for the creation of comprehensive scientific learning activities addressing difficult scientific topics, and it was successfully used in both presential and remote learning. Furthermore, Plant Ecology students were enthusiastic about the experience, as it eased their process of learning about plants. Several of them acknowledged gaining a higher awareness of plants in their daily lives and a deeper connection with them, and some have also become regular users of iNaturalist in their free times.

Apps and citizen science platforms are remarkable tools for connecting students and the public with nature and engaging society in environmental challenges such as biodiversity conservation. Many authors have emphasized that formal education alone is insufficient to alter people’s views and behaviours towards conservation^31^. They have identified the “extinction of experience”—Pyle’s term for the growing alienation between people and nature—as the primary cause of this lack of public awareness of the biodiversity crisis^32,33^. The activities here described boosted the interest and motivation of those involved towards nature, particularly plants. This interaction promotes nature experience, sharpens observational skills, deepens understanding of how nature works and, ultimately, strengthens emotional and mental ties to nature^34^. Allowing students to distract from the COVID-imposed restrictions, also helped maintain their mental health^35,36^.

The incorporation of new technologies into the teaching of botany and plant ecology, as an addition to or substitute for the more formal approaches, is also an opportunity to engage the younger generations, known as digital natives (Box 2). Using their proximity to these instruments helps both to stimulate the interest in plant sciences and to combat the escalating lack of enthusiasm in this important science field. Additionally, apps and citizen science are “cool” activities that attract the attention of media and institutions, amplifying the impact of these actions^37^.

The use of these technologies raises several issues (Box 2). First, general inertia dominates schools and universities^38^, so overcoming the “business as usual” resistance in teaching botany is critical to include alternative teaching techniques that involve a higher degree of commitment. In fact, for the students, teachers, and scientists who support these initiatives, activities typically take more time than traditional methods (e.g., conducting fieldwork, and validating findings), so an extra effort from all involved is required. Moreover, there may be challenges associated with tech-literacy limits for use and economical constraints for smartphone use^39,40^. Last, but not least, ethical issues surrounding data sharing must be considered, such as the location of endangered species and private student information, which are currently being debated^41,42^.

The analysis of our results revealed a significant bias in the participants’ profile. In fact, a small group of people recorded most of the observations, in a remarkable approximation to the Pareto principle (which states that 80% of the results result from 20% of the causes). This trend was reinforced in the formal education activities when a few enthusiastic students provided significantly more observations than those requested in the activities. Much engagement work is therefore required to increase and sustain participation in citizen science programs^27^. To do so, it is necessary to understand what motivates people to sign up for these programs, encourage their participation, and go beyond those who are already engaged with it.

To summarize, our work supports that citizen science and mobile apps have a high potential for boosting interest in plants, both in formal and informal learning activities. The tools used were able to provide answers to relevant plant ecology questions. Also, were equally successful in raising awareness of the importance of the native Flora in a broader context near the general public and university students. In the face of an unprecedented biodiversity crisis, citizen science and apps are therefore excellent tools for addressing societal engagement in biodiversity conservation and environmental issues.

Box 2 Pros, cons and challenges of using Citizen Science and Mobile Apps at formal education

**Table:** 

Pros	Cons	Challenges
• Encourage students’ engagement • Promote scientific literacy • It is “cool”: encourage Media and Institutional engagement	• Time consuming for students, teachers, and scientists • Potential age and financial constrains	• Overcoming the inertia of formal education • Ethical concerns about data sharing, including private and sensitive information (such as students and protected species location). • Surmount the Pareto principle

### Reporting summary

Further information on research design is available in the Nature Research Reporting Summary linked to this article.

## Supplementary information

Reporting Summary

Supplementary Data 1

Supplementary Data 2

Supplementary Data 3

Supplementary Data 4

## Data Availability

The datasets generated and/or analysed during the current study are available in the iNaturalist/Biodiversity4All platform or included as Supplementary information.
