# Supplementary material for: Rescuing Botany: using citizen-science and mobile apps in the classroom and beyond
Source: NPJ Biodivers. 2023 Mar 1;2:6. doi: 10.1038/s44185-023-00011-9 (PMC9975877; doi:10.1038/s44185-023-00011-9)
Supplement: Supplementary file 2 — Supplementary Data 1 [file 44185_2023_11_MOESM2_ESM.docx]

# **Supplementary Data 1 Fieldwork Protocol for the Ecology practical classes**

This protocol includes all methods required to accomplish the objectives of Activity 1: Analysis of the impact of disturbance on plant diversity in grasslands, specifically the selection of sampling sites and plots and how to characterise them based on the level of perturbation found, all sampling methods for plants, pollinators, and soil, a manual to identify and register plants, and, finally, how to use the iNaturalist/Biodiversity4All platform and Flora-on web.

Fieldwork Protocol for the Ecology practical classes

Part A (2020/2021)

Version 1.0

Alice Nunes^1^, Helena Serrano^1^, Fernando Ascensão^1^, Sergio Chozas ^1^ e Cristina Branquinho^1^

1 - Centre for Ecology, Evolution and Environmental Changes (cE3c) - FCUL, Portugal


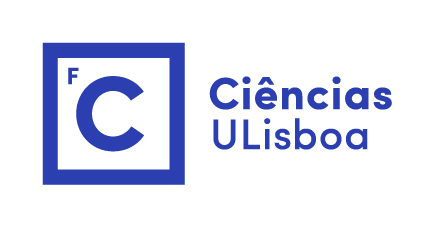

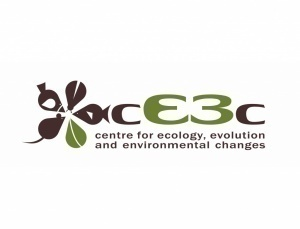


15/02/2021

This is a description of the field work to be developed in Part A of the Ecology discipline. Point 1 should be completed during the first practical class (from 15 to 19^th^ February). Points 2 to 5 should be done at the same time (during the same field visit), before the second practical class (PL2 from 22 to 26^th^ February), to which students should bring the soil samples.

**1. Selection of the sampling site(s)**

Each student should select one or more sampling sites in the surroundings of her/his area of residence, which may be a **Disturbed**, **Semi-natural** or **Natural** green area. These areas should have preferably at least 1 ha (100 x 100 m) and include grassland patches with well-developed herbaceous vegetation. These may include urban parks, gardens, uncultivated areas, open woodlands, or other natural ecosystem types. Each site (or sites) selected by each student will be a replicate of the “Disturbance level” treatment (low, medium, or high). Before or during the first practical class (Feb 15-19), each student should register the location of the selected site(s) by creating a [kmz](https://developers.google.com/kml/documentation/kmzarchives?hl=pt-PT) (<https://developers.google.com/kml/documentation/kmzarchives?hl=pt-PT>) file ([using google earth](https://developers.google.com/kml/documentation/kmzarchives?hl=pt-PT), see [here](https://www.ccdr-a.gov.pt/docs/upload/REN/KML.pdf) (https://www.ccdr-a.gov.pt/docs/upload/REN/KML.pdf) for details on how to create a kml/kmz file) and taking some photographs of the site (e.g., Fig. 1). In each site, 3 types of sampling procedures will be carried out, focused on i) the herbaceous vegetation ii) pollinators and iii) soil. All data will be stored in a unique shared file (Google forms; see below). We will then analyze the effect of disturbance and site characteristics (e.g., soil) on the composition and diversity of herbaceous plants and associated pollinators.


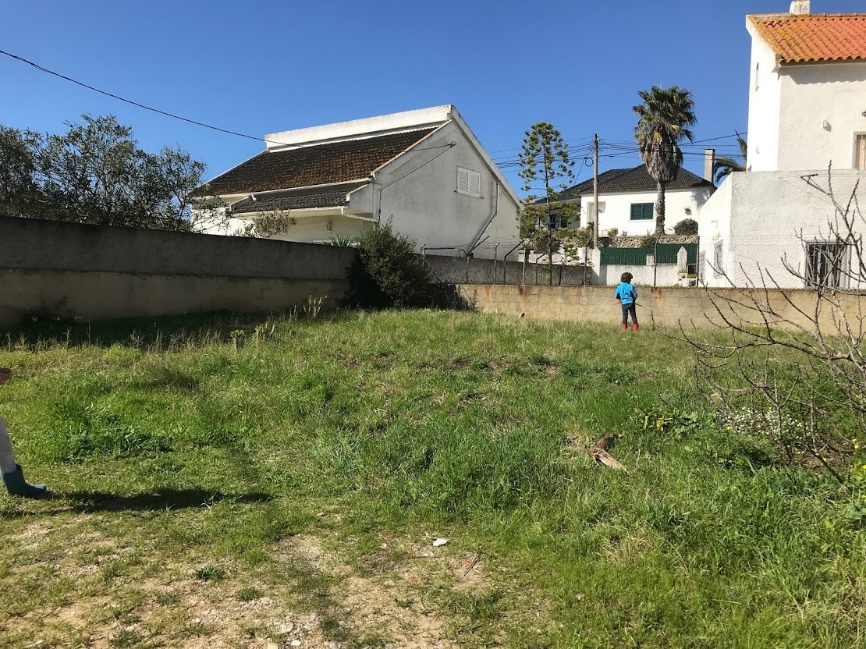


**Figure 1.** Ruderal community of herbaceous species in a suburb area

**2. Definition of the sampling plots on each site and screening of the main herbaceous species**

At each site, each student should define three 1 x 1 meter plots, which correspond to the **sample unit** (see Fig. 2). These plots should be placed in homogeneous areas dominated by herbaceous vegetation (excluding woody species such as shrubs or trees), focusing on flowering species, to facilitate their identification.

KML file of the site and photographs of both site and plots should be uploaded to the folder [KML file and photographs Folder - Site and plots](https://moodle.ciencias.ulisboa.pt/mod/assign/view.php?id=140500), located in the Moodle platform.


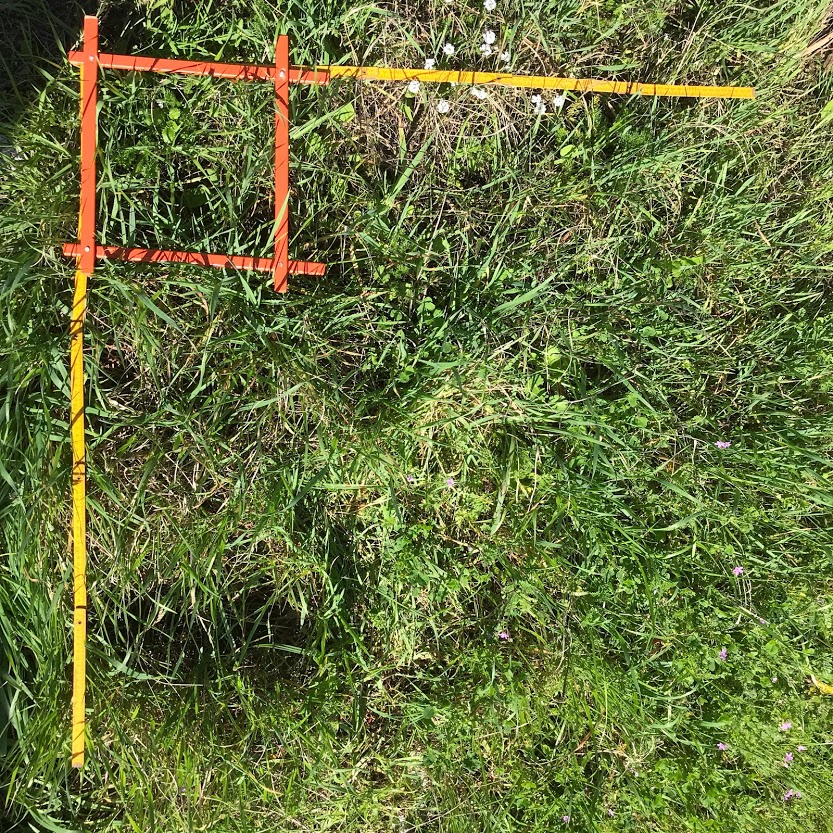


**Figure 2.** Sample unit - 1 x 1 m plot with a 25 x 25 cm quadrat. We used a wooden folding rule for the plot and a wooden quadrat, but feel free to use anything you have at home.

We advise using the [iNaturalist](https://www.inaturalist.org/home) (<https://www.inaturalist.org/home>) application and websites suggested in the classes, mainly [flora-on](https://flora-on.pt/index.php) (https://flora-on.pt/index.php), to help in the identification of the herbaceous species present on your site(s). IMPORTANTLY, all **flower species** present in your Sample Units must be submitted to iNaturalist/Biodiversity4all in the [***Ecologia2_FCUL***](https://www.biodiversity4all.org/projects/ecologia2_fcul) (<https://www.biodiversity4all.org/projects/ecologia2_fcul>) project (see Annex I on how to use iNaturalist). This will help you to correctly identify the species, with the assistance of Prof. Sergio.

**3. Sampling pollinators**

After defining each of the 1 x 1 m plots, students should look for pollinators inside the plot (1 x 1 m) (I.e., **before collecting the information on flowers and soil**). It may be helpful to delimit the plot with a line or by other means, to facilitate the detection of the insects inside the Sampling Unit. **Prior** to starting the counting of pollinators, you should step back at least 1 meter from the Sampling Unit and wait 2 minutes quietly (and who is with you also). You will then record all the insects standing on the flowers (resting or feeding) inside the Sampling Unit. The survey will take 10 minutes.

You will then record the total number of insects per group (Fig. 3), within the Sampling Unit. For example, if you see 1 bee and then 1 butterfly, and then another bee and 2 beetles, and finally 2 more butterflies, all landing/inspecting the flowers inside your Sampling Unit, during the 10 min. Survey, your input data in the [Google](https://docs.google.com/forms/d/16XdFfhHUALtie-XgGMMGcam3ld7HDbbRE_MhVFDFrwU/viewform?edit_requested=true) form will be: bees 2; butterflies 3; beetles 2; **flies 0** [**one row per taxon**]. Repeat the process for each Sampling Unit. Also take note of the date and time you started the survey (HH:MM); wind conditions (no wind, medium, high) and temperature (check you phone or meteo.pt).


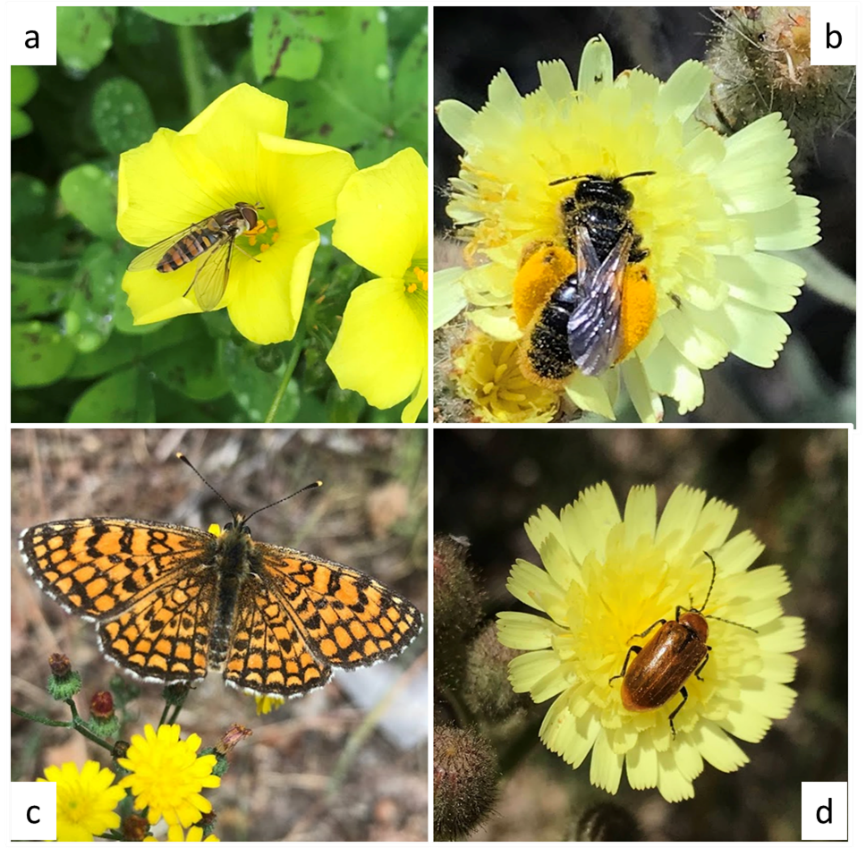


Figure 3: Pollinator groups selected for the study a) flies (Diptera), b) bees (Hymenoptera),
c) butterflies (Lepidoptera) and d) beetles (Coleoptera)

**4. Herbaceous vegetation sampling**

The sampling of vegetation aims to characterize the dominant herbaceous species and estimate (roughly) their cover. At each site, each 1 x 1 m plot should be divided into 16 smaller quadrats with 25 x 25 cm (Figure 2 and 4). To define the plot and grid in place, you can use the materials that seem most suitable to you, for example, measuring tapes, 1 meter rod, 50 x 50 cm or 25 x 25 cm “frames”, or others. Within each plot, all flowering species present should be registered, as well as the number of small quadrats in which it is present (Figure 4). The registration can be done on a field sheet (Annex 2) or directly (or afterwards) inserted through this [Google form](https://docs.google.com/forms/d/14L1Ta6wLLzGbcgE_74RXE00OrTRiUhWlUseiwJFJ1W4/viewform?edit_requested=true).


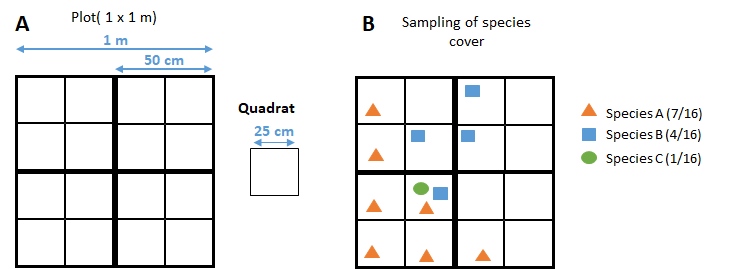


**Figure 4.** A) Sampling unit: 1 x 1 m plot, divided into 16 smaller 25 x 25 cm quadrats; B) example of species presence counting in the quadrats.

The cover of each species will be estimated by calculating the sum of the number of quadrats (25 x 25 cm) where it is present, divided by the total number of quadrats sampled (in our case, 16). Subsequently, we will use the number of species, their characteristics (e.g., type of flower) and the respective cover to calculate metrics of functional and taxonomic diversity.

**5. Soil sampling**

Collect soil into a plastic bag, mixing all the samples well into the same bag = **1 composite soil sample per site**. Avoid the areas you plan to sample for vegetation, but go around it doing a zig-zag walk and collecting soil at each step. You should not go deeper than 15 cm, because that is the zone where most plants have roots, and most nutrients are located. Remove plants, roots, or stones from the composite soil sample. The number of steps/sub-samples should be at least five, up to 15. The total volume of soil collected should be enough to fill 3 cups, that you will use during the Laboratorial class (total ca. 1 Liter of soil). Take a **picture** of the soil and annotate if it was **dry or wet from rain or from irrigation**, and **any other pertinent observation**.


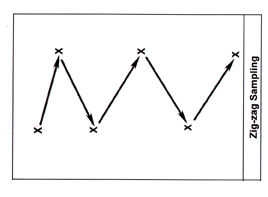
------>
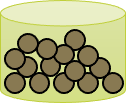


Back at home, take a quick look at the soil collected. Do you see any **living beings (macro-megafauna)**? If yes, take a **picture**, annotate **how many of each type/species** and release the animals back into nature. Then **leave the plastic bag open, for the soil to air dry** until the Laboratorial class (if too wet, leave it in a warm division of the house to dry).

During the class, soil data will be inserted in an excel file (see ex.Table 2 - complete excel template will be available in Moodle – Activity: **PL2 - Pratica Laboratorial 2 - soils**), photographs will be uploaded to a **Soil Photographs Folder** in Moodle (Activity: **PL2 - Photographs Folder - soils**) and final results through a [Google form](https://forms.gle/7q1PZUkoz3mKmi3fA) in Moodle (Activity: Link **PL2 - Soil Form to insert the common results for the Project**).

**Table 2**. Example of soil table to fill for the soil data. *Depending on the amount of macro-megafauna that the students find, later it may be asked to include it in the iNaturalist App. At the moment that is not possible, so please save the pictures.

| **Sample ID** | **GPS** | **Collection Date** | **Notes** | **Photos** |
| --- | --- | --- | --- | --- |
| **19163** | 38.764, -9.155 | 2021-02-17 | **Slightly wet soil**, from irrigation (it is a municipal garden but with spontaneous plants growing. It might have been fertilized. It is irrigated once a week.) | 19163_soil.jpg |
| TASK 1 (BIODIVERSITY) | | | | |
| **Species/types*** | **Number of individuals found** | | **Photo ref.** | |
| #01 ants | 1 | | 19163_spp_01.jpg | |
| *#02 marias-café* | 3 | | 19163_spp_02.jpg | |
| #03 worms | 3 | | 19163_spp_03.jpg | |
| #04 unknown Spp. | 1 | | 19163_spp_04.jpg | |
| #05 unknown Spp. | 2 | | 19163_spp_05.jpg | |
| ... | ... | | ... | |
|  | | | | |

**Annex 1 How to register observations on iNaturalist/BioDiversity4All platform.**


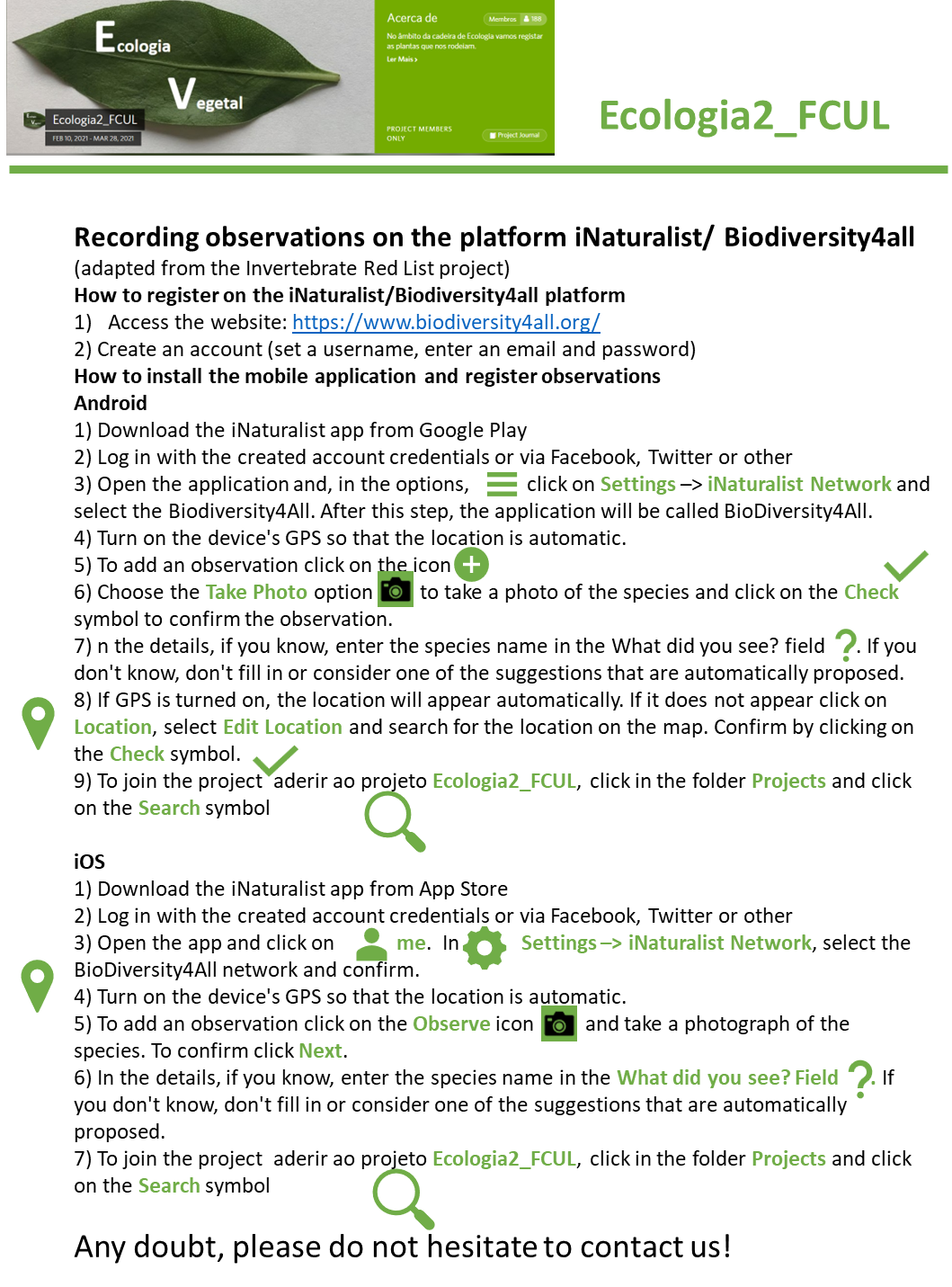


**Annex 2 - Fieldwork sheets (Ecology 2020/2021)**

**(Use one sheet per Sampling Unit)**

**Student name____________________ Student number___________**

**Site ______________ Sampling Unit_________ Date__________ Hour _________**

**Temperature ____________ Wind ___________**

**Pollinators**

| Pollinator taxa | Count | Observations (genera, species, etc.; others) | Photo |
| --- | --- | --- | --- |
| Butterflies |  |  |  |
| Flies |  |  |  |
| Beetles |  |  |  |
| Bees |  |  |  |
|  |  |  |  |
|  |  |  |  |
|  |  |  |  |
|  |  |  |  |
|  |  |  |  |
|  |  |  |  |
|  |  |  |  |
|  |  |  |  |

**If you can identify the species or genera, use the blank rows to record. But in Google Sheet jut insert the totals for each of the four main groups.**

**Herbaceous**

|  | Number of quadrats | | |  |
| --- | --- | --- | --- | --- |
| **Species** | **Plot 1** | **Plot 2** | **Plot 3** | **Observations** |
|  |  |  |  |  |
|  |  |  |  |  |
|  |  |  |  |  |
|  |  |  |  |  |
|  |  |  |  |  |
|  |  |  |  |  |
|  |  |  |  |  |
|  |  |  |  |  |
|  |  |  |  |  |
|  |  |  |  |  |
|  |  |  |  |  |
|  |  |  |  |  |
|  |  |  |  |  |
|  |  |  |  |  |
|  |  |  |  |  |
|  |  |  |  |  |
|  |  |  |  |  |
|  |  |  |  |  |
|  |  |  |  |  |
|  |  |  |  |  |
|  |  |  |  |  |
|  |  |  |  |  |
|  |  |  |  |  |
|  |  |  |  |  |
|  |  |  |  |  |
|  |  |  |  |  |
|  |  |  |  |  |
|  |  |  |  |  |
