# Supplementary material for: Rescuing Botany: using citizen-science and mobile apps in the classroom and beyond
Source: NPJ Biodivers. 2023 Mar 1;2:6. doi: 10.1038/s44185-023-00011-9 (PMC9975877; doi:10.1038/s44185-023-00011-9)
Supplement: Supplementary file 3 — Supplementary Data 2 [file 44185_2023_11_MOESM3_ESM.docx]

# **Supplementary Data 2 - Assessing the specific leaf area (SLA)**

This appendix includes all methods required to accomplish the objectives of Activity 2: Assessing the specific leaf area (SLA), specifically how to select the plant species and collect, weight, measure the area - utilising mobile apps-, and dry the leaves to determine the SLA of each species.

Specific leaf area (SLA) is the ratio of leaf area to leaf dry mass and indicates how much leaf area a plant builds with a given amount of leaf biomass.

**SLA= A/M_L_,**

where **A** is the area of a set of leaves and M_L_ is the dry mass of those leaves. Typical units are m^2^/kg or mm^2^/mg.

Drought and water stress have varying effects on specific leaf area. Species with typically low specific leaf area values are geared for the conservation of acquired resources, due to their large dry matter content, high concentrations of cell walls and secondary metabolites, and high leaf and root longevity. Additionally, interspecific variations are found in a variety of species. Under drought conditions, leaves of certain species are smaller than leaves on watered plants. In fact, decrease in surface area would mean that there would be fewer ways for water to be lost.

**Objective**

The objective of this work will be the assessment of the SLA of two or three shrub or tree species. Ideally each species will belong to a different functional group regarding their adaptation to water, namely Hydrophytes, Mesophytes and Xerophytes. You can select any species occurring near your place such as *Olea europaea,* *Nerium oleander, Phillyrea angustifolia*, *Hydrangea macrophylla*, etc. or, even better, you can join the *Quercus* challenge!


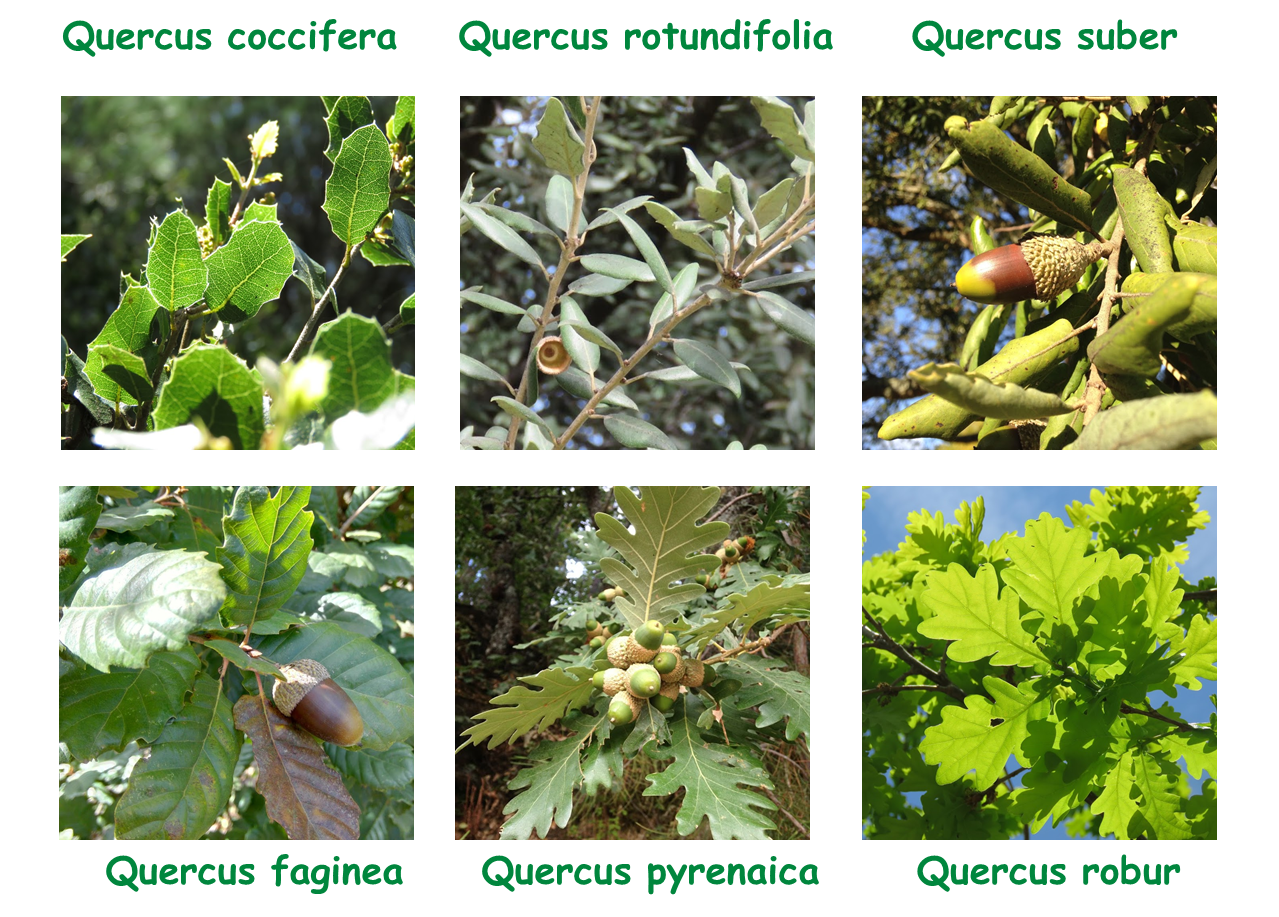


Figure 1. Quercus species sorted according to their tolerance to aridity (higher tolerance, left-up, lower tolerance right-down)

***Quercus* challenge**

In Portugal, *Quercus* genus is composed by [several species](https://flora-on.pt/#/1quercus) (<https://flora-on.pt/#/1quercus>; +/- 8 taxonomy in progress). They occur in nearly all shrub and forest habitats throughout the country. Some species, such as *Quercus coccifera* or *Q. rotundifolia,* tolerate high aridity levels while others such as *Q. robur* live in mesophytic forests. So, this genus constitutes a perfect plant group to compare SLA values and, therefore, we propose you to assess the SLA of at least two *Quercus* species.

**Methods (for all species, including *Quercus*)**

1.- At least 30-40 leaves of each species will be sampled, depending on the size and the weight of the leaves and on the accuracy of your kitchen scale (Figure 2)


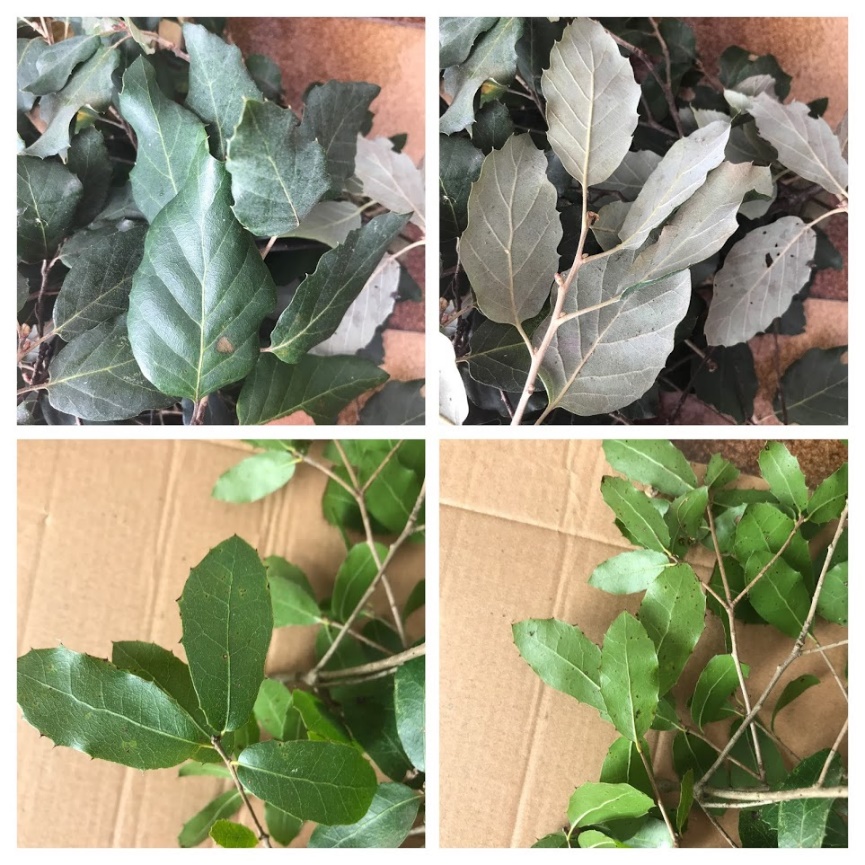


Figure 2 – Leaves of *Quercus suber* (up) and *Q. coccifera* (down).

2.-Leaf area (individually and using mobile app for apple: [LeafByte](https://zoegp.science/leafbyte), <https://zoegp.science/leafbyte> or android: <Leaf-it>, <https://play.google.com/store/apps/details?id=com.heaslon.EasyLeafArea&hl=pt_PT&gl=US>) and weight (all leaves together using your kitchen scale) will be measured after collecting (Figure 3 up) Accuracy of kitchen scales is normally low so a considerable number of leaves will be necessary to properly weight the leaves.

If you have enough leaves, you can divide your leaves into 3 groups to calculate the average and standard deviation.


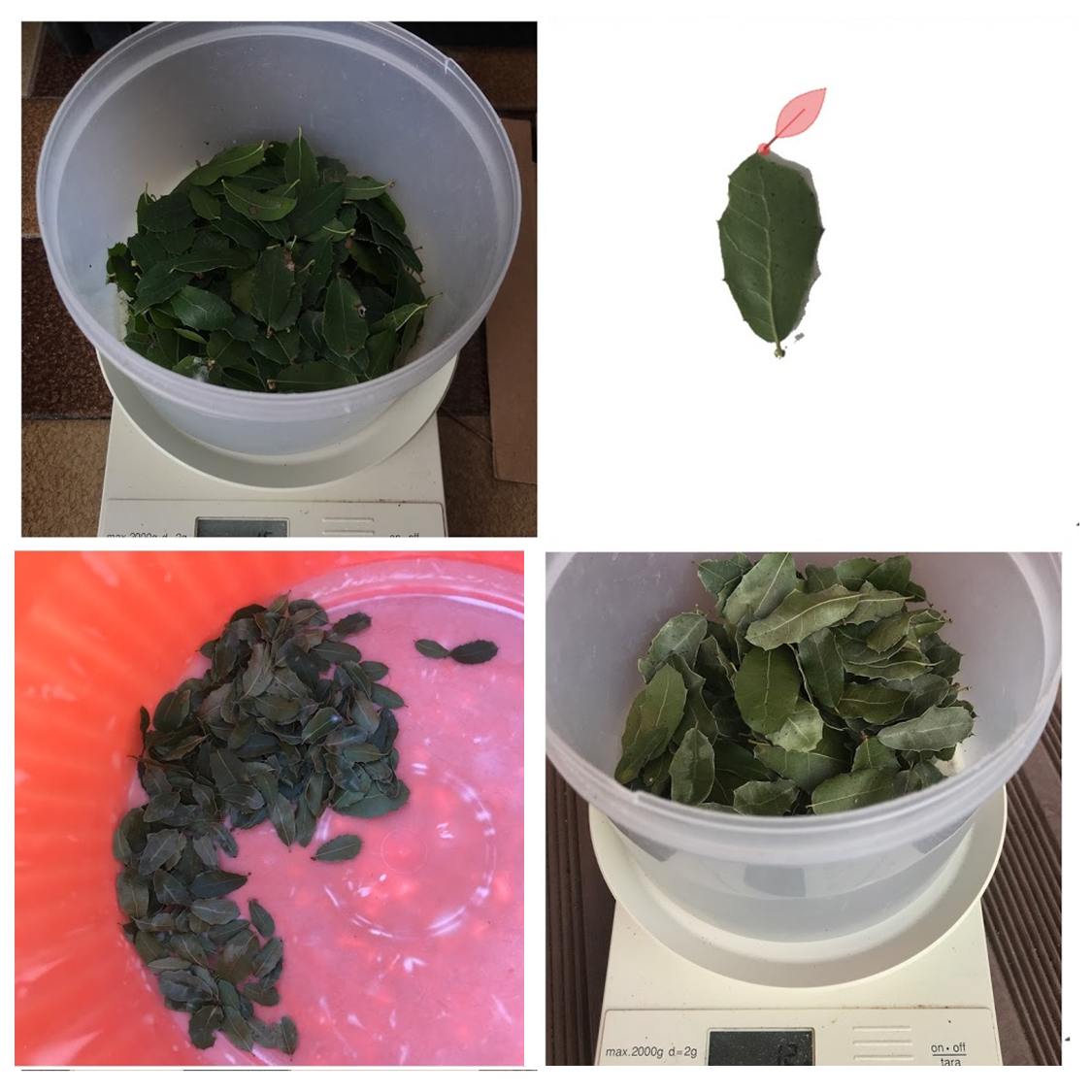


Figure – Leaves will be i) weight after collecting, ii) then their area will be measured, iii) dried (sun or microwaves oven) and iv) weight again when totally dried.

3. Leaves will be dried (sun or microwave oven) and then weight again (Figure 3, down) to calculate SLA (see formula).

Tips for drying leaves in the microwave oven:

- Place a small bowl of water in the microwave for safety and place leaves in a folded paper towel. Weigh down with a microwave-able plate.
- Microwave on high for 30 seconds and continue in 15 second intervals until the leaves are dry and crisp. **NOTE:** leaves can catch on fire in the microwave, so it is important you only microwave in these short bursts and that you have the bowl of water in the microwave as well to absorb some of the heat.
- Please note that microwave ovens vary in temperature controls. You may find that a high setting burns your leaves, so you might have to experiment with a medium or low heat setting.

Here you can find a [YouTube tutorial](https://www.youtube.com/watch?v=b797pvRouZ0) (https://www.youtube.com/watch?v=b797pvRouZ0)

4. Finally, we can also calculate the water content of our leaves following the formula bellow:

Water content= (weight_fresh_ – weight_dry_ / weight_fress_)*100
