# Supplementary material for: Rescuing Botany: using citizen-science and mobile apps in the classroom and beyond
Source: NPJ Biodivers. 2023 Mar 1;2:6. doi: 10.1038/s44185-023-00011-9 (PMC9975877; doi:10.1038/s44185-023-00011-9)

# **Supplementary Data 3 - SLA, LMA and WC assessment and** **Quercus challenge results** Plant Ecology – 2nd year 20/21

| **Species** | **Records** | **SLA*** | **SD** | **LMA*** | **SD** | **WC*** | **SD** |
| --- | --- | --- | --- | --- | --- | --- | --- |
| *Acacia longifolia* | 4 | 4.67 | 2.46 | 94.98 | 68.00 | 56.55 | 11.02 |
| *Acacia pycnantha* | 2 | 6.06 | 0.48 | 86.63 | 97.55 | 61.25 | 1.77 |
| *Acacia saligna* | 1 | 6.99 |  | 143.14 |  | 64.29 |  |
| *Acacia sp.* | 1 | 14.62 |  | 68.36 |  | 57.14 |  |
| *Acer pseudoplatanus* | 1 | 9.73 |  | 1.03 |  | 64.29 |  |
| *Aeonium arboreum* | 1 | 4.50 |  | 22.22 |  | 73.91 |  |
| *Aesculus hippocastanum* | 1 | 4.89 |  | 204.34 |  | 73.33 |  |
| *Agapanthus praecox* | 1 | 4.20 |  | 23.72 |  | 85.00 |  |
| *Aloe arborescens* | 1 | 1.09 |  | 92.04 |  | 84.97 |  |
| *Arbutus unedo* | 8 | 8.19 | 83.92 | 1.97 | 67.09 | 61.83 | 7.90 |
| *Arundo donax* | 1 | 6.86 |  | 14.57 |  | 18.75 |  |
| *Atriplex halimus* | 1 | 14.47 |  | 69.11 |  | 76.47 |  |
| *Atriplex portulacoides* | 1 | 1.03 |  | 97.00 |  | 73.30 |  |
| *Buxus sempervirens* | 1 | 3.92 |  | 25.54 |  | 0.60 |  |
| *Callistemon citrinus* | 1 | 1.38 |  | 853.50 |  | 44.70 |  |
| *Camellia rosiflora* | 1 | 1.60 |  | 626.40 |  | 55.00 |  |
| *Carpobrotus edulis* | 2 | 1.57 | 1.09 | 22.49 | 30.11 | 47.05 | 65.68 |
| *Carylus avellana* | 1 | 2.59 |  | 0.39 |  | 0.50 |  |
| *Castanea sativa* | 1 | 12.53 |  | 20.51 |  | 61.11 |  |
| *Ceratonia siliqua* | 1 | 3.50 |  | 28.57 |  | 33.00 |  |
| *Ceratonia siliqua* | 1 | 3.08 |  | 32.52 |  | 20.00 |  |
| *Citrus reticulata* | 1 | 3.56 |  | 28.06 |  | 29.41 |  |
| *Citrus sinensis* | 3 | 10.21 | 8.46 | 159.09 | 123.80 | 71.16 | 10.39 |
| *Citrus x limon* | 1 | 4.68 |  | 217.63 |  | 46.43 |  |
| *Cotoneaster coriaceus* | 2 | 6.70 | 0.28 | 14.16 | 0.48 | 36.88 | 51.09 |
| *Crassula sp.* | 1 | 3.80 |  | 261.00 |  | 0.90 |  |
| *Crataegus monogyna* | 1 | 14.69 |  | 34.00 |  | 50.00 |  |
| *Cydonia oblonga* | 3 | 31.44 | 28.75 | 22.57 | 31.13 | 65.91 | 6.41 |
| *Cyperus involucratus* | 1 | 12.80 |  | 78.00 |  | 60.40 |  |
| *Cyperus sp.* | 1 | 1.43 |  | 697.70 |  | 40.00 |  |
| *Echeveria elegans* | 1 | 1.47 |  | 678.89 |  | 75.00 |  |
| *Eichhornia crassipes* | 1 | 9.77 |  | 102.31 |  | 87.50 |  |
| *Eleagnus reflexa* | 1 | 4.17 |  | 239.00 |  | 45.45 |  |
| *Eriobotrya japonica* | 7 | 3.50 | 1.86 | 393.72 | 457.71 | 41.22 | 21.86 |
| *Eucalyptus globulus* | 8 | 3.80 | 1.96 | 204.91 | 157.85 | 43.56 | 18.30 |
| *Eucalyptus leucoxylon* | 1 | 4.19 |  | 2.39 |  | 24.14 |  |
| *Eucalyptus spp.* | 1 | 5.82 |  | 171.79 |  | 18.00 |  |
| *Ficus benghalensis* | 1 | 1.95 |  | 512.16 |  | 0.70 |  |
| *Ficus benjamina* | 1 | 7.84 |  | 127.55 |  | 37.50 |  |
| *Ficus carica* | 11 | 8.15 | 5.35 | 104.60 | 100.00 | 7209.76 | 23707.00 |
| *Ficus macrophylla* | 1 | 4.74 |  | 210.78 |  | 65.45 |  |
| *Fraxinus angustifolia* | 5 | 10.93 | 7.28 | 3638.88 | 8092.98 | 43.61 | 39.33 |
| *Hedera sp.* | 4 | 10.61 | 8.23 | 393.91 | 625.58 | 49.84 | 33.60 |
| *Hibiscus palustris* | 1 | 6.00 |  | 162.79 |  | 80.18 |  |
| *Hydragea macrophylla* | 15 | 15.61 | 7.19 | 62.99 | 75.29 | 63.37 | 34.09 |
| *Lantana camara* | 1 | 3.79 |  | 264.00 |  | 8.97 |  |
| *Laurus nobilis* | 4 | 5.36 | 1.35 | 196.23 | 51.26 | 15.19 | 17.08 |
| *Laurus nobilis* | 1 | 4.96 |  | 201.56 |  | 70.00 |  |
| *Ligustrum lucidum* | 5 | 7.62 | 1.66 | 54.52 | 59.80 | 52.22 | 6.83 |
| *Lonicera periclymenum* | 2 | 14.25 | 0.00 | 38.61 | 44.68 | 32.83 | 45.50 |
| *Magnolia grandiflora* | 1 | 2.24 |  | 447.10 |  | 53.00 |  |
| *Malus domestica* | 1 | 51.04 |  | 1959.00 |  | 14286.00 |  |
| *Manilkara zapota* | 1 | 0.87 |  | 1107.92 |  | 59.00 |  |
| *Merendera montana* | 1 | 7.29 |  | 13.20 |  | 62.50 |  |
| *Metrosideros excelsa* | 2 | 11.15 | 0.21 | 88.30 | 0.27 | 42.80 | 13.86 |
| *Morus alba* | 1 | 15.7 | 6.37 |  |  | 71.43 |  |
| *Myoporum acuminatum* | 1 | 7.51 |  | 13.08 |  | 66.81 |  |
| *Myoporum laetum* | 4 | 3.89 | 2.68 | 197.95 | 250.93 | 56.10 | 11.88 |
| *Myrtoideae* | 1 | 3.45 |  | 2.90 |  | 29.73 |  |
| *Nelumbo nucifera* | 1 | 11.21 |  | 89.17 |  | 82.28 |  |
| *Nerium oleander* | 28 | 4.44 | 196.39 | 2.57 | 149.79 | 46.21 | 23.89 |
| *Olea europaea* | 59 | 4.96 | 220.75 | 3.05 | 479.06 | 37.10 | 21.11 |
| *Parthenocissus tricupidata* | 1 | 26.70 |  | 37.40 |  | 80.88 |  |
| *Pelargonium cacullatum* | 1 | 1.30 |  | 76.92 |  | 63.00 |  |
| *Phyllostachys aurea* | 1 | 17.34 |  | 57.67 |  | 71.40 |  |
| *Phytolacca heterotepala* | 1 | 18.56 |  | 53.89 |  | 0.85 |  |
| *Pitto sporum undulatum* | 1 | 7.64 |  | 130.95 |  | 59.26 |  |
| *Pittosporum tobira* | 1 | 3.64 |  | 27.47 |  | 0.46 |  |
| *Platanus hispanica* | 3 | 9.56 | 5.14 | 117.24 | 123.91 | 48.42 | 42.12 |
| *Platanus mexicana* | 1 | 12.54 |  | 79.69 |  | 0.61 |  |
| *Platanus orientalis* | 1 | 30.73 |  | 14.66 |  | 71.82 |  |
| *Prunus cerasifera* | 1 | 11.01 |  | 9.08 |  | 79.49 |  |
| *Prunus cerasus* | 1 | 27.37 |  | 36.54 |  | 62.50 |  |
| *Prunus dulcis* | 3 | 6.81 | 3.69 | 48.39 | 43.35 | 55.70 | 15.85 |
| *Prunus laurocerasus* | 3 | 12.41 | 14.35 | 31.94 | 12.58 | 58.69 | 16.05 |
| *Prunus lusitanica* | 1 | 6.03 |  | 166.00 |  | 61.90 |  |
| *Prunus sp.* | 2 | 7.59 | 6.77 | 58.23 | 31.90 | 40.56 | 55.97 |
| *Quercus coccifera* | 11 | 5.58 | 3.87 | 159.48 | 155.26 | 24.94 | 22.55 |
| *Quercus faginea* | 10 | 13.59 | 12.77 | 85.31 | 38.74 | 51.95 | 23.27 |
| *Quercus ilex* | 3 | 5.72 | 6.80 | 403.78 | 298.31 | 56.04 | 15.71 |
| *Quercus imbricaria* | 1 | 10.32 |  | 95.65 |  | 60.00 |  |
| *Quercus lusitanica* | 1 | 0.99 |  | 100.96 |  | 56.00 |  |
| *Quercus polymorpha* | 1 | 22.00 |  | 4.55 |  | 80.00 |  |
| *Quercus pyrenaica* | 2 | 11.94 | 3.46 | 87.44 | 25.32 | 69.67 | 0.94 |
| *Quercus robur* | 11 | 18.62 | 11.27 | 55.85 | 42.37 | 75.11 | 12.50 |
| *Quercus rotundifolia* | 12 | 7.98 | 2.77 | 128.56 | 63.64 | 47.85 | 19.71 |
| *Quercus sp.* | 3 | 17.07 | 5.20 | 38.18 | 27.70 | 47.96 | 42.69 |
| *Quercus suber* | 34 | 8.80 | 6.11 | 104.40 | 84.66 | 43.81 | 19.99 |
| *Rhamnus alaternos* | 4 | 6.16 | 3.19 | 150.84 | 93.40 | 50.83 | 6.14 |
| *Rumex crispus* | 1 | 12.00 |  | 83.36 |  | 78.00 |  |
| *Ruscus aculeatus* | 1 | 8.69 |  | 25.30 |  | 54.55 |  |
| *Salix alba* | 1 | 7.54 |  | 132.59 |  | 50.00 |  |
| *Salix laevigata* | 1 | 4.58 |  | 218.27 |  | 0.29 |  |
| *Salix sp.* | 1 | 20.35 |  | 49.14 |  | 75.00 |  |
| *Schefflera arboricola* | 2 | 17.92 | 5.03 | 174.47 | 148.35 | 78.01 | 7.48 |
| *Smilax aspera* | 2 | 13.72 | 0.00 | 40.10 | 46.39 | 33.89 | 46.97 |
| *Strelitzia reginae* | 1 | 2.21 |  | 97.31 |  | 53.66 |  |
| *Suculenta* | 1 | 8.60 |  | 116.10 |  | 94.00 |  |
| *Teucrium fruticans* | 1 | 7.67 |  | 130.43 |  | 0.24 |  |
| *Vibrurnum tinus* | 5 | 4.48 | 1.88 | 29.19 | 31.37 | 22.70 | 15.27 |
| *Vinca difformis* | 2 | 4.41 | 178.21 | 0.71 | 35.79 | 72.92 | 14.73 |
| *Washingtonia robusta* | 1 | 1.76 |  | 567.10 |  | 38.00 |  |
| *Yucca elephantipes* | 1 | 0.60 |  | 1655.46 |  | 0.13 |  |
| *Zantedeschia aethiopica* | 1 | 1.16 |  | 865.50 |  | 0.60 |  |

SLA (mm2/mg)

LMA  (g/m2)

Page Break

***Quercus* challenge**


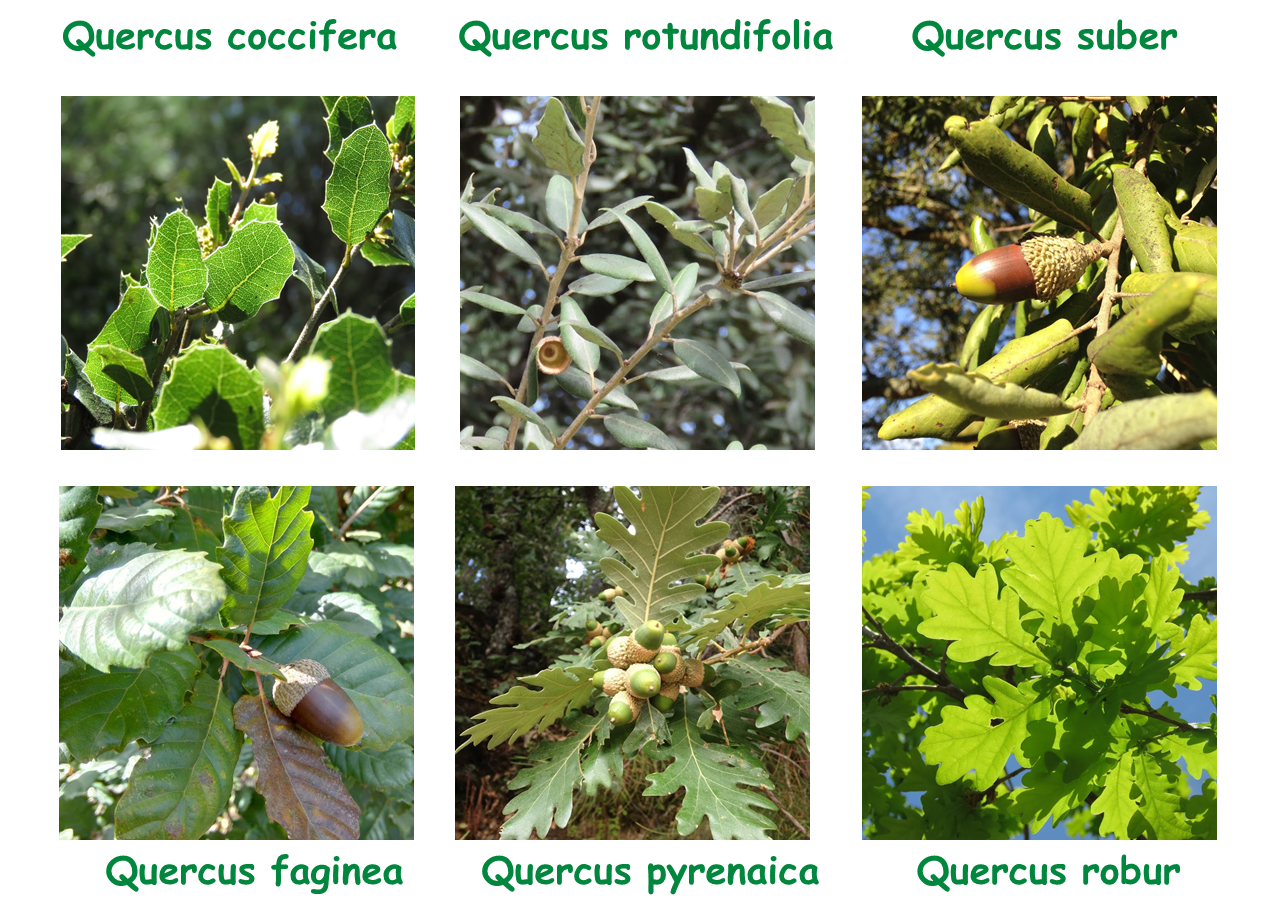


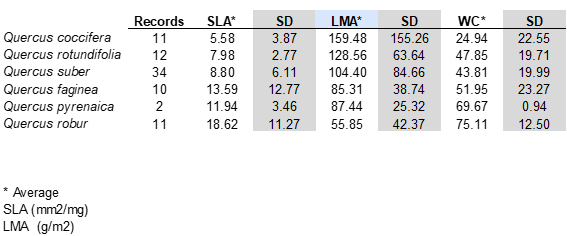

Supplement: Supplementary file 4 — Supplementary Data 3 [file 44185_2023_11_MOESM4_ESM.docx]
