# Supplementary material for: Rescuing Botany: using citizen-science and mobile apps in the classroom and beyond
Source: NPJ Biodivers. 2023 Mar 1;2:6. doi: 10.1038/s44185-023-00011-9 (PMC9975877; doi:10.1038/s44185-023-00011-9)

# **Supplementary Data 4 - Guidelines for sampling the herbaceous and pollinator communities of four lawns at the University of Lisbon** Plant Ecology –2021/2022

The main objective of the sampling will be to analyse if the intensity in the use and management of the lawns of the University of Lisbon condition i) the specific and functional richness (traits) of the herbaceous community of the same and ii) the provision of ecosystem services of these lawns, in this case of pollination.

To this end, we will characterize the herbaceous vegetation and pollinators of four lawns located at the University of Lisbon (Figure 1), and formulate the following more specific questions:

1. How does the specific diversity of herbs and pollinators respond to the management of the sampled lawns?
2. Is there a relationship between the diversity of herbaceous species and that of pollinators?
3. And between the structural complexity of the herbaceous community and the diversity of pollinators?
4. How does the functional diversity of herbs respond to lawn management?
5. Is there a relationship between the diversity of pollinators and the functional diversity of herbs

The answers to these questions may indicate management strategies to maximize the diversity of herbaceous and pollinator species as well as their functional diversity and the provision of ecosystem services.


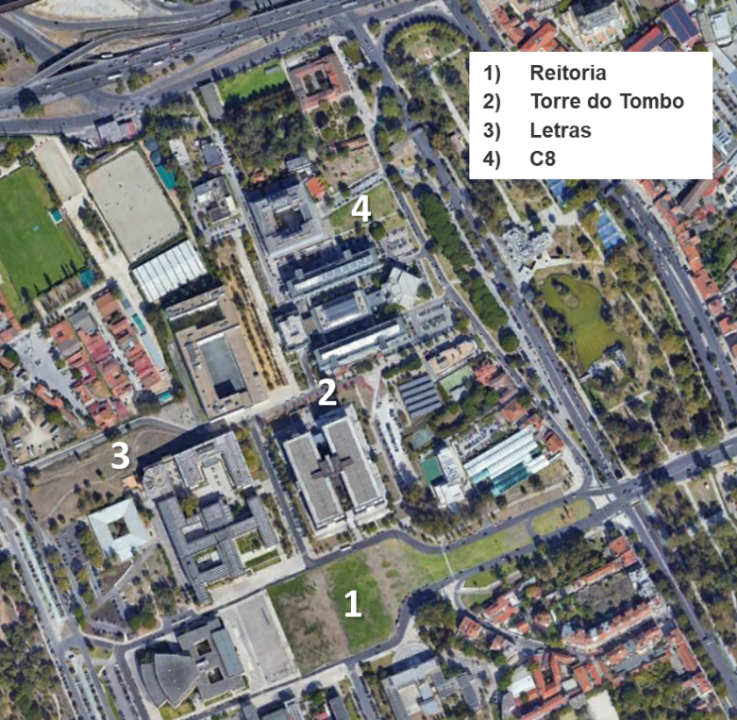


Figure 1 – Sampling sites

**Methods**

All lawn will be sampled by 3 groups, except the *Relvado* (lawn) *da Reitoria* which will be sampled by 4 groups. Each group will be subdivided into 4 subgroup and each group will sample a total of 3 transects of 10 m, making a total of 36 transects/lawn (3 groups x 4 subgroups x 3 transects), except in the *Reitoria*, where there will be 48 transects (4 groups x 4 subgroups x 3 transects). The lawns were divided into 10x10 m square grids (Figure 2) in order to facilitate a homogeneous distribution of the sampling sites. Each group will locate its 3 transects within one of the squares indicated by the responsible teacher (Figure 3).

| 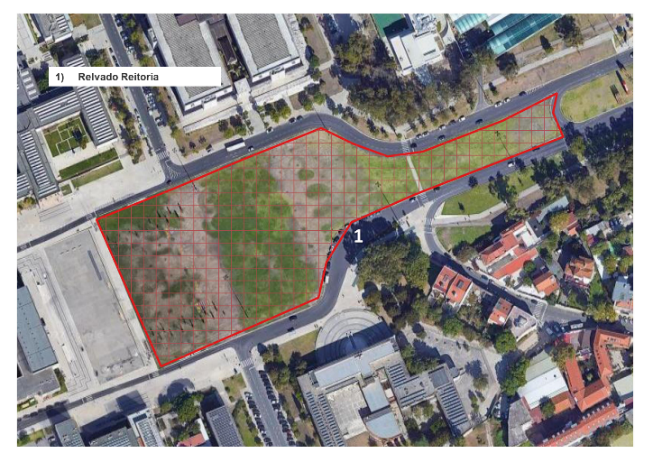 | 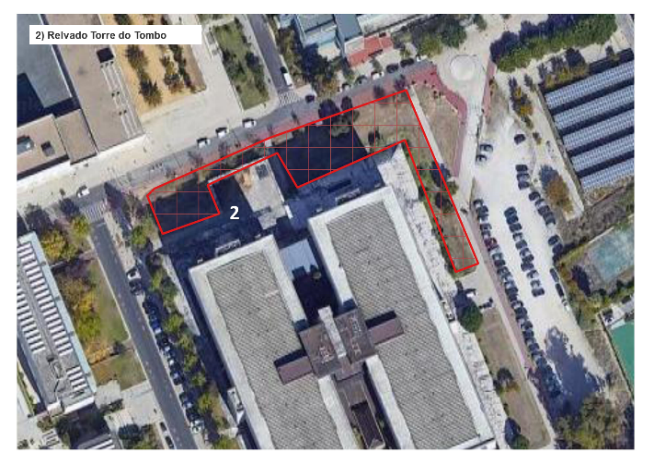 |
| --- | --- |
| **Reitoria** | **Torre do Tombo** |
| 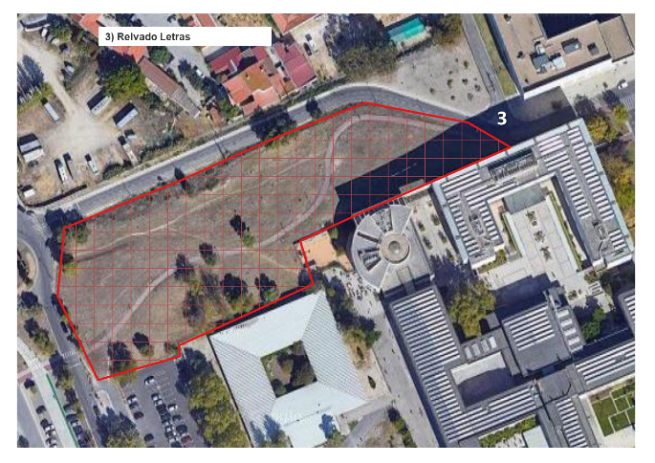 | 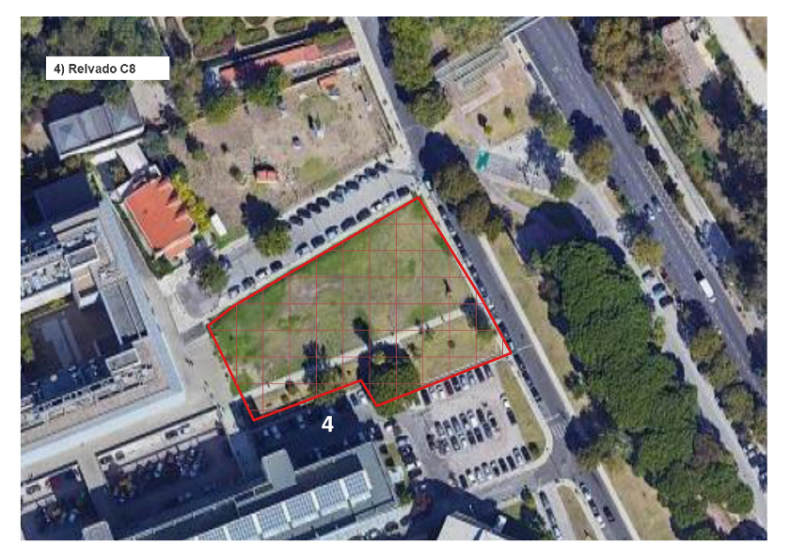 |
| **Letras** | **C8** |

Figure 2 – Sampling grids.


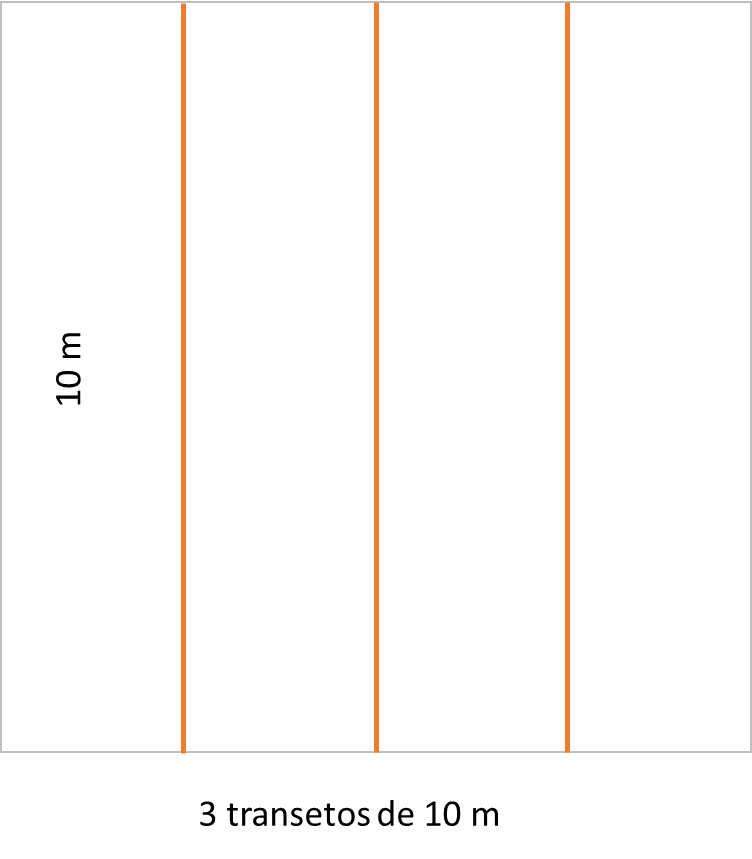


Figure 3 – Transect distribution within the 10x10 m square.


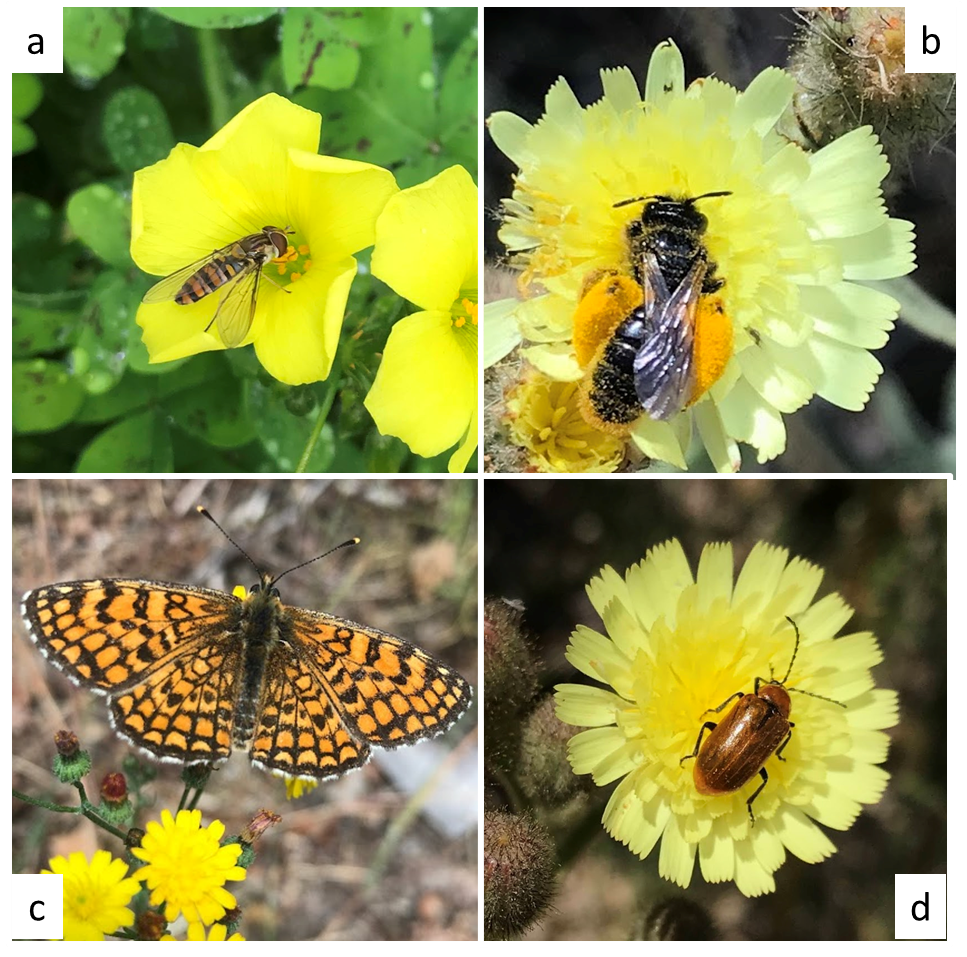


Figure 4 – Most common pollinators in Portuguese lawns: a) flies (Diptera), b) bees (Hymenoptera), c) butterflies (Lepidoptera) e d) beetles (Coleoptera).

In order to minimize the effect of the disturbance on pollinating insects, we will first proceed to characterize them: the number of pollinators (bees, butterflies, flies, etc. see Figure 4 or https://www.life4pollinators.eu/index.php/en/downloads) will be recorded for 5 minutes at the beginning (0 m), center (5 m) and end (10 m) of each of the transects.

The coverage of the herbaceous community will be characterized using the Point-Intercept Sampling Method (Figure 5). This method consists of recording the species of each individual (herbaceous) that touches a wand arranged perpendicularly along the transect of 10 m every 50 cm. For each species, the total coverage will be equal to the number of “touches” divided between the maximum possible number (in this case 20, because 0 will not be counted). The height of individuals who touch the wand will also be recorded.

Before and during sampling, students will use the iNaturalist application to help identify herbaceous species and pollinators present in the transect, and register them in the [**Ecologia 2 Relvados 2022**](https://www.biodiversity4all.org/projects/ecologia-2-relvados-2022) (<https://www.biodiversity4all.org/projects/ecologia-2-relvados-2022>) project (see Annex I). The species found will be recorded on a field sheet (Annex II).

| 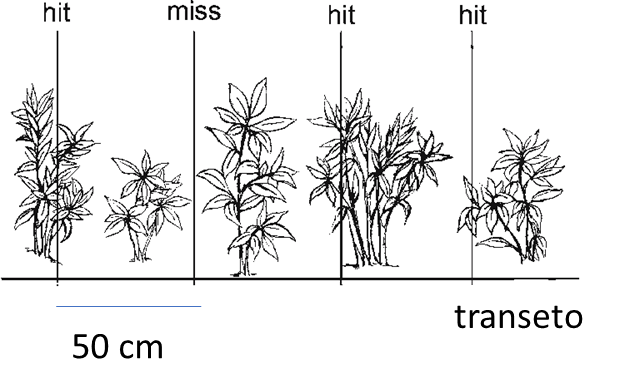 | 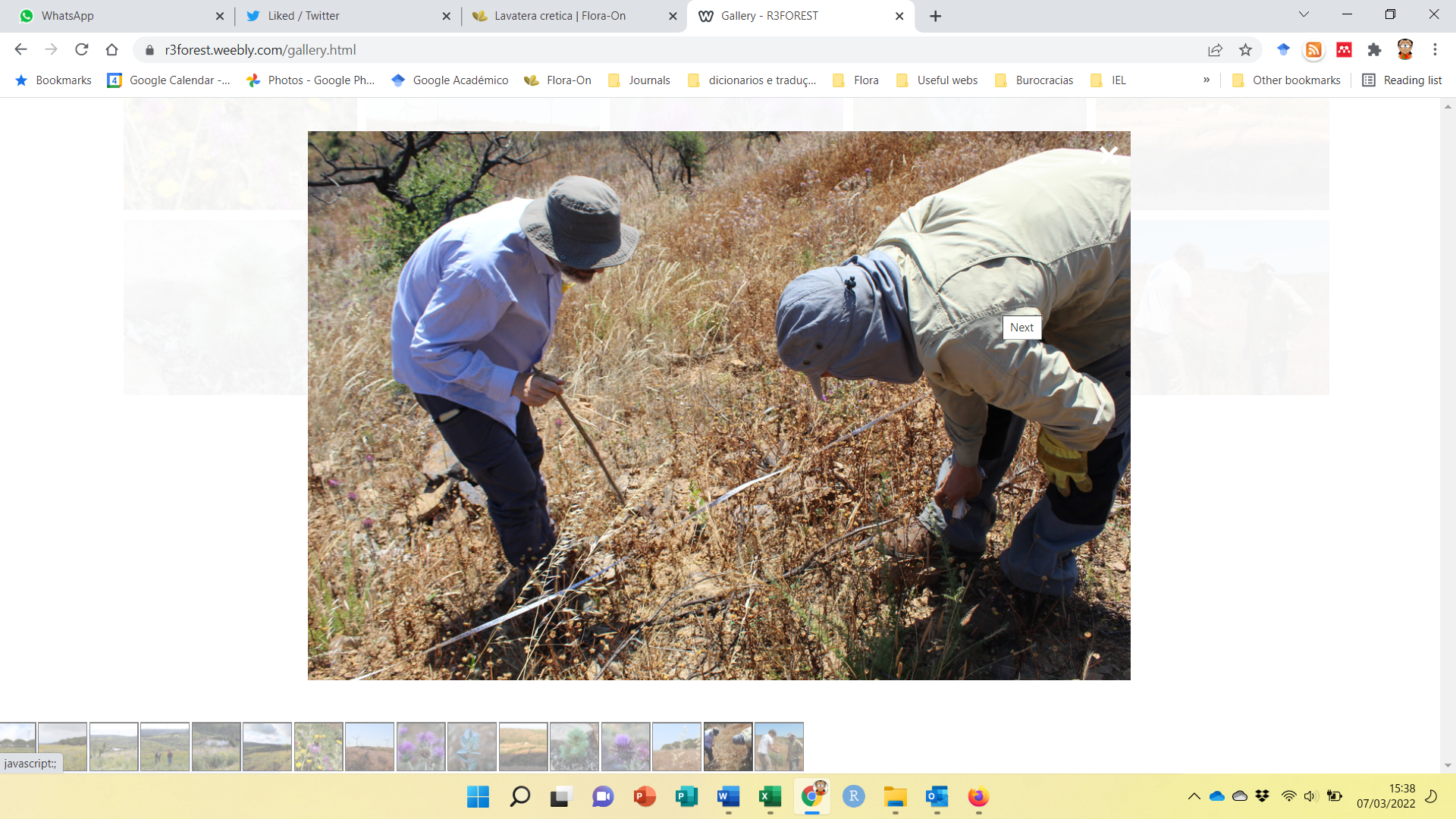 |
| --- | --- |

Figure 5 – Point-Intercept Sampling Method

**Annex I – Field sheet**

**Annex II – How to register data in the iNaturalist/BioDiversity4All platform**


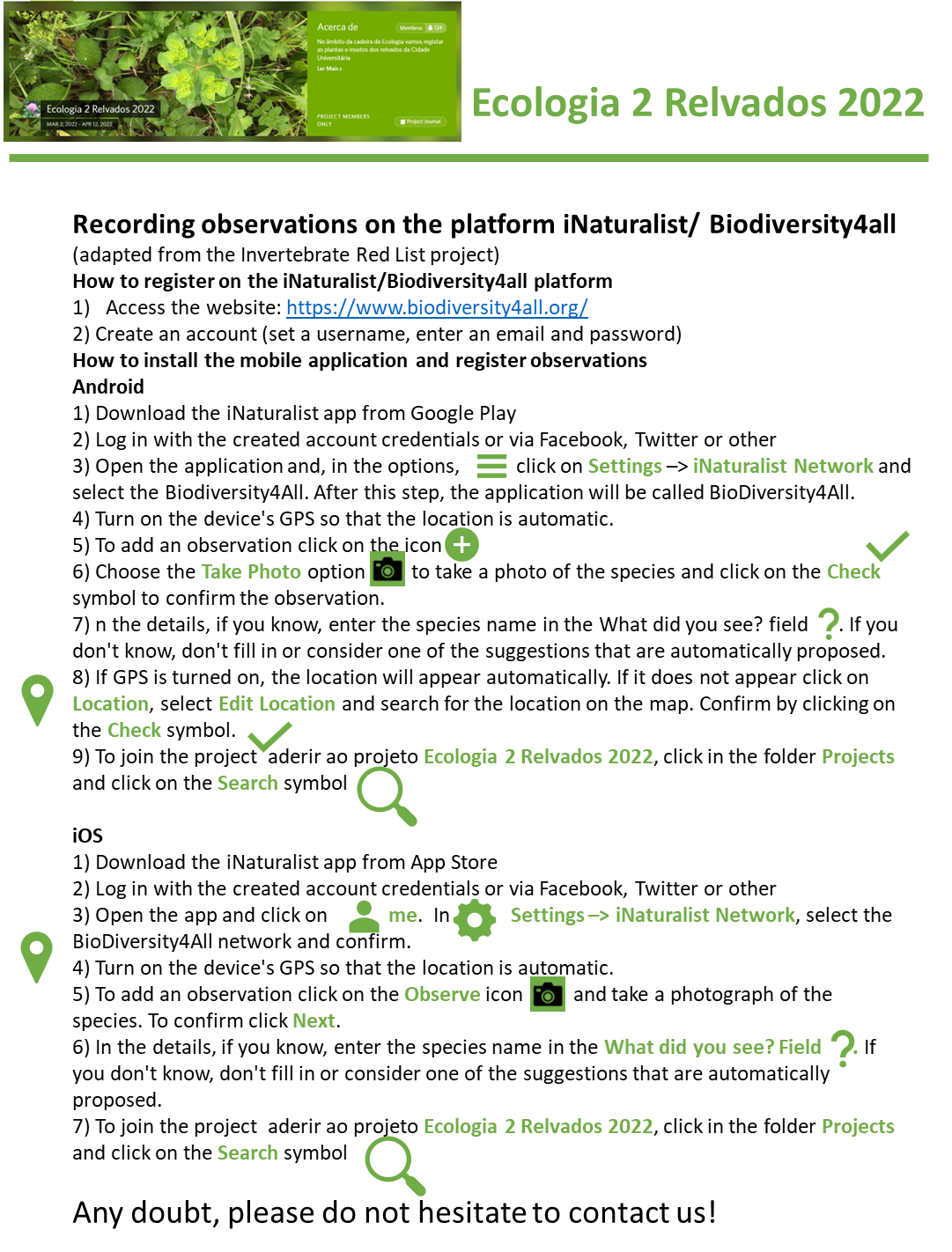

Supplement: Supplementary file 5 — Supplementary Data 4 [file 44185_2023_11_MOESM5_ESM.docx]
